# A newly developed and externally validated non-clinical score accurately predicts 10-year cardiovascular disease risk in the general adult population

**DOI:** 10.1038/s41598-021-99103-4

**Published:** 2021-10-04

**Authors:** Catarina Schiborn, Tilman Kühn, Kristin Mühlenbruch, Olga Kuxhaus, Cornelia Weikert, Andreas Fritsche, Rudolf Kaaks, Matthias B. Schulze

**Affiliations:** 1 Department of Molecular Epidemiology, German Institute of Human Nutrition Potsdam-Rehbruecke (DIfE), Nuthetal, Germany; 2 German Center for Diabetes Research (DZD), Munich Neuherberg, Germany; 3 Division of Cancer Epidemiology, German Cancer Research Center (DKFZ), Heidelberg, Germany; 4 Institute for Global Food Security, Queen’s University Belfast, Belfast, UK; 5 Department of Food Safety, German Federal Institute for Risk Assessment, Berlin, Germany; 6 Institute for Social Medicine, Epidemiology and Health Economics, Charité University Medical Center, Berlin, Germany; 7 Institute for Diabetes Research and Metabolic Diseases of the Helmholtz Center Munich at the University of Tübingen, Tübingen, Germany; 8 Division of Endocrinology, Diabetology, Vascular Disease, Nephrology and Clinical Chemistry, Department of Internal Medicine, University of Tübingen, Tübingen, Germany; 9 Institute of Nutritional Science, University of Potsdam, Nuthetal, Germany; 10 Department of Molecular Epidemiology, German Institute of Human Nutrition Potsdam-Rehbruecke (DIfE), Arthur-Scheunert-Allee114-116, 14558 Nuthetal, Germany

**Keywords:** Cardiovascular diseases, Disease prevention, Prognosis, Epidemiology, Population screening

## Abstract

Inclusion of clinical parameters limits the application of most cardiovascular disease (CVD) prediction models to clinical settings. We developed and externally validated a non-clinical CVD risk score with a clinical extension and compared the performance to established CVD risk scores. We derived the scores predicting CVD (non-fatal and fatal myocardial infarction and stroke) in the European Prospective Investigation into Cancer and Nutrition (EPIC)-Potsdam cohort (n = 25,992, cases = 683) using competing risk models and externally validated in EPIC-Heidelberg (n = 23,529, cases = 692). Performance was assessed by *C*-indices, calibration plots, and expected-to-observed ratios and compared to a non-clinical model, the Pooled Cohort Equation, Framingham CVD Risk Scores (FRS), PROCAM scores, and the Systematic Coronary Risk Evaluation (SCORE). Our non-clinical score included age, gender, waist circumference, smoking, hypertension, type 2 diabetes, CVD family history, and dietary parameters. *C*-indices consistently indicated good discrimination (EPIC-Potsdam 0.786, EPIC-Heidelberg 0.762) comparable to established clinical scores (thereof highest, FRS: EPIC-Potsdam 0.781, EPIC-Heidelberg 0.764). Additional clinical parameters slightly improved discrimination (EPIC-Potsdam 0.796, EPIC-Heidelberg 0.769). Calibration plots indicated very good calibration with minor overestimation in the highest decile of predicted risk. The developed non-clinical 10-year CVD risk score shows comparable discrimination to established clinical scores, allowing assessment of individual CVD risk in physician-independent settings.

## Introduction

Cardiovascular diseases (CVD) are a major public health burden^1^. Prognostic CVD prediction models allow identifying individuals at high risk that are eligible for lifestyle interventions and preventive treatment by estimating individual CVD risk. Their development is largely focussed on applications in clinical settings to support treatment decisions as for example with the Systematic COronary Risk Evaluation (SCORE) and the Pooled Cohort Equations (PCE)^2–5^. However, as these evaluations require information from physical examinations (blood pressure) and blood tests (cholesterol), application of these scores is unfeasible in most physician-independent settings like self-assessment of individuals, health education campaigns, and step-wise screening procedures including a non-clinical stage. The few available non-clinical models to be used independently of physical examinations are limited in terms of study design, originating from case–control studies or high-risk cohorts^6,7^; short follow-ups and lack of equations to calculate absolute risks^6,7^; the endpoints, predicting only myocardial infarction (MI) or stroke^7,8^; or inclusion of dietary predictors on a nutrient level requiring assessment of a large variety of individual foods, thus hampering the applicability in practice^6,9^. We only identified one model allowing large-scale estimation of individual CVD risk based on non-clinical parameters^10^. However, despite established risk associations, the score does not include potentially informative dietary information^11^.

Moreover, overlap in risk factor profiles of CVD and type 2 diabetes (T2D) offers the potential for combined risk assessment with only minor deviations in the required predictors, including dietary parameters. The German Diabetes Risk Score (GDRS) is a multiply validated non-clinical score to predict T2D and its extension for CVD risk prediction would enable simultaneous quantification of individual CVD and T2D risk in non-clinical settings^12^.

Thus, we aimed to develop and externally validate a non-clinical risk score to predict 10-year CVD risk based on shared predictors with the GDRS and to compare its performance to the identified non-clinical and established clinical CVD risk scores. Furthermore, we developed a clinical extension with routinely available clinical predictors for step-wise screening approaches.

## Results

Descriptive comparison of the unimputed and imputed data, including the proportion of missingness, is presented in the supplement (Supplementary Table (ST) 1). The median follow-up time in the European Prospective Investigation into Cancer and Nutrition (EPIC)-Potsdam was 11.35 years (interquartile range (IQR) 1.38). Both samples contained proportionally more women than men (female EPIC-Potsdam: 61.6%; EPIC-Heidelberg: 54.6%) and the median age at baseline was 50 years (Table 1). Prevalence of self-reported hypertension was higher in Potsdam (31.8%) compared to Heidelberg (27.2%), while the proportion of participants reporting a family history of CVD was higher in Heidelberg (52.8%, Potsdam: 37.1%), as well as current heavy smoking (≥ 20 units/day) at baseline (Potsdam 5.7%, Heidelberg 9.5%).

**Table 1:** Baseline characteristics of the EPIC-Potsdam and EPIC-Heidelberg cohorts.

Parameter	EPIC-Potsdam n = 25,993	EPIC-Heidelberg n = 23,529
	Median (IQR) or %	
Age at baseline [years]	50 (16)	50 (14)
Gender (female)	61.6%	54.7%
Waist circumference [cm]	85.0 (19.0)	87.9 (19.5)
Physical activity [h/week]	4.5 (6.5)	2.3 (3.0)
**Smoking status**		
Never	47.7%	42.0%
Former [< 20 units/d]	23.0%	19.8%
Former [≥ 20 units/d]	8.9%	14.5%
Current [< 20 units/d]	14.8%	14.1%
Current [≥ 20 units/d]	5.7%	9.6%
**Family history of CVD**		
One parent diseased	29.5%	40.3%
Both parents diseased	4.4%	7.8%
At least one sibling diseased	6.4%	12.6%
**Self-reported diabetes at baseline**	4.2%	3.0%
**Self-reported hypertension at baseline**	31.8%	27.2%
**Usual dietary intake**		
Whole grains [50 g portion/d]	0.6 (1.3)	0.8 (1.4)
Plant oil [10 g portion/d]	0.2 (0.3)	0.6 (0.4)
Coffee [150 ml portion/d]	2.0 (2.6)	2.0 (3.3)
High-energy soft drinks [200 ml portion/d]	0.01 (0.12)	0.02 (0.16)
Red meat [150 g portion/d]	0.2 (0.2)	0.2 (0.3)
**Systolic BP [mmHg]**	126.8 (22.0)	126.5 (22.0)
**Diastolic BP [mmHg]**	82.5 (14.0)	82.0 (14.3)
**Triglycerides [mg/dl]**	107.8 (83.1)	141.7 (106.3)
**Total cholesterol [mg/dl]**^a^	200.3 (52.7)	228.2 (54.1)
**HDL-cholesterol [mg/dl]**^a^	53.1 (18.1)	54.1 (21.3)
**HbA**_**1c**_ [%]	5.5 (0.7)	5.4 (0.5)

IQR, interquartile range. CVD, cardiovascular disease. BP, blood pressure. HDL, high density lipoprotein. HbA_1c_, glycated haemoglobin.

^a^to convert cholesterol to mmol/L, multiply values by 0.0259.

### Score derivation

The final non-clinical model included the predictors age, gender, waist circumference, smoking status, self-reported hypertension and T2D, CVD family history, and consumption of whole grain, red meat, coffee, high energy soft drinks, and plant oil. The clinical model additionally contained systolic and diastolic blood pressure, total and HDL cholesterol.

The proportional hazards assumption was fulfilled for all included predictors. The supremum test for functional form was only significant for ‘CVD points’ in the clinical model. However, subsequent examination of the according restricted cubic splines did not indicate strong deviations from a linear function (Supplementary Figure (SF) 1).

Estimates derived by using Cox proportional hazards regression and the Fine and Gray model were overall comparable. However, comparison of the model performance indicated slightly better calibration of absolute risks by the Fine and Gray model compared to the Cox model in the upper risk range (SF2). As a consequence, we proceeded with the competing risk approach.

Adding statistically significant interaction terms or squared terms as well as deriving gender-specific equations of the Fine and Gray models did not improve overall performance relevantly (SF3).

The final parameters used for absolute risk calculation based on the competing risk model are depicted in Table 2 (example calculation: Supplementary Note (SN) 1).

**Table 2:** Risk associations of the included predictors with CVD and parameters used for absolute risk calculation.

Risk predictors	sHR	Coefficients^a^	Score points^b^
**Non-clinical score**			
Age at baseline [years]	1.08	0.076455	7.6
Gender (male)	1.83	0.605795	61
Waist circumference [cm]	1.01	0.008732	1
Former smoker [< 20 units/d]	0.95	− 0.04816	− 5
Former smoker [≥ 20 units/d]	1.10	0.094238	9
Current smoker [< 20 units/d]	2.02	0.702442	70
Current smoker [≥ 20 units/d]	2.98	1.090835	109
Self-reported hypertension	1.62	0.481992	48
Self-reported diabetes	1.58	0.456607	46
One parent with CVD	1.51	0.411488	41
Both parents with CVD	1.80	0.588969	59
At least one sibling with CVD	1.93	0.656639	66
Whole grains [50 g portion/d]	0.90	− 0.106495	− 11
Red meat [150 g portion/d]	1.43	0.357274	36
Coffee [150 g portion/d]	0.97	− 0.034486	− 3
High-energy soft drinks [200 ml portion/d]	1.07	0.072234	7
Plant oil [10 g portion/d]	0.88	− 0.129972	− 13
Subdistribution baseline survival *S*_*0*_^c^			0.98614
Baseline Σ_i_β_i_ $\overline{{\mathrm{X}}}$_i_^d^			517.766
**Clinical score**			
Points non-clinical score	2.445	0.894039	0.89
Systolic BP [mmHg]	1.006	0.005662	0.57
Diastolic BP [mmHg]	1.013	0.012542	1.25
Total cholesterol [mg/dl]	1.004	0.003825	0.38
HDL-cholesterol [mg/dl]	0.995	− 0.004806	− 0.48
Subdistribution baseline survival *S*_*0*_^c^			0.98683
Baseline Σ_i_β_i_ $\overline{{\mathrm{X}}}$_i_^d^			691.006

Risk associations are mutually adjusted and depicted as hazard ratios.

sHR, subdistribution hazard ratio. CVD, cardiovascular disease. BP, blood pressure. HDL, high density lipoprotein.

^a^β coefficients estimated by Cox proportional hazards regression.

^b^β coefficients were rounded and multiplied by 100 to derive according score points for absolute risk calculation.

^c^*S*_*0*_, baseline subdistribution survival used to calculate absolute risk.

^d^Σ_i_β_i_ $\overline{{\mathrm{X}}}$_i_, mean score points of all participants at baseline.

### Performance in EPIC-Potsdam and EPIC-Heidelberg

#### Discrimination

Competing risk-adjusted *C*-indices indicated good discrimination of both developed models in EPIC-Potsdam (non-clinical: 0.786, 95% confidence interval (95%CI) 0.736–0.832; clinical 0.796, 0.746–0.841) and EPIC-Heidelberg (non-clinical: 0.762, 0.715–0.807; clinical: 0.769, 0.721–0.813). The categorical Net-Reclassification-Improvement (NRI) suggested only slight improvement of risk category assignment by additional clinical parameters (NRI EPIC-Potsdam: 0.015, 95%CI − 0.028 to 0.057; EPIC-Heidelberg 0.078, 0.041–0.116). Sensitivity and specificity in both cohorts are shown in the ST2. As an example, when using a cut-off of 5% predicted risk in EPIC-Heidelberg, sensitivity and specificity were 48.8% and 83.4% for the non-clinical and 53.3% and 81.9% for the clinical score.

**Figure 1:**
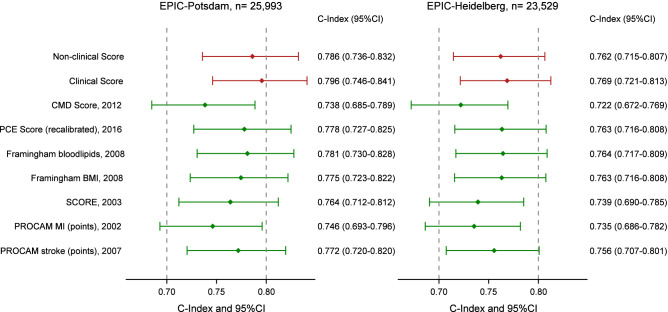
Discrimination of the developed scores and established CVD risk scores in EPIC-Potsdam and EPIC-Heidelberg. Discrimination is depicted as *C*-indices adjusted for competing risk analyses and 95% confidence intervals (95%CI). EPIC, European Prospective Investigation into Cancer and Nutrition. CMD, chronic metabolic disease. BMI, body mass index. MI, myocardial infarction. PCE, Pooled Cohort Equation. SCORE, Systematic Coronary Risk Evaluation.

Comparison of the performance with established risk scores demonstrated that the two derived equations reached the highest *C*-indices in EPIC-Potsdam (e.g., Framingham CVD Risk Score (FRS) with blood lipids 0.781, 0.730–0.828) (Fig. 1). In EPIC-Heidelberg, *C*-indices were overall slightly lower than in EPIC-Potsdam. The *C*-index of the non-clinical score ranged among the highest, comparable to established clinical scores (e.g., FRS with blood lipids 0.764, 0.717–0.809), while the derived clinical score still showed the highest *C*-index. *C*-indices of the non-clinical chronic metabolic disease (CMD) score were considerably lower in EPIC-Potsdam (0.738, 0.685–0.789) and EPIC-Heidelberg (0.722, 0.672–0.769).

#### Calibration

**Figure 2:**
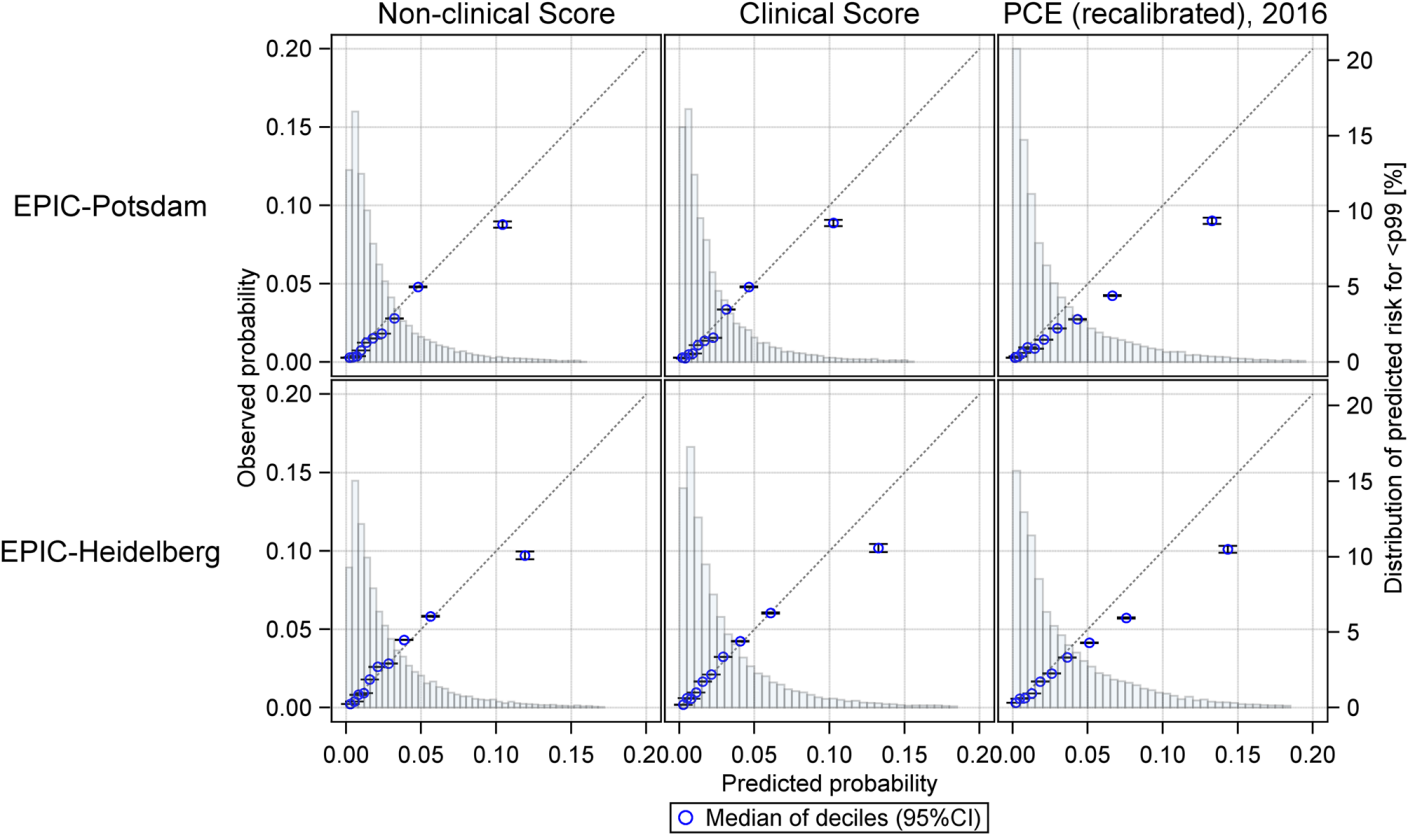
Calibration plots for the developed scores and the recalibrated PCE in EPIC-Potsdam and EPIC-Heidelberg. Observed and predicted CVD risk is grouped by deciles of predicted risk and plotted with the according 95% confidence interval (95%CI). Distribution of predicted risk up to the 99th percentile (p) is indicated in the background. EPIC, European Prospective Investigation into Cancer and Nutrition. PCE, Pooled Cohort Equation.

The derived scores were well calibrated for the majority of individuals in the lower nine deciles of predicted risk while they slightly overestimated risk in the highest decile of predicted risk (Fig. 2). Expected-to-observed ratios were 1.17 (95%CI 1.08–1.27) for the non-clinical and 1.13 (1.04–1.22) for the clinical score in EPIC-Potsdam and 1.05 (0.97–1.13) and 1.11 (1.03–1.20) in EPIC-Heidelberg, respectively. Calibration plots suggested slight overestimation of risk by the recalibrated PCE (Fig. 2) and substantial overestimation by both FRS (not shown).

### Subgroup and sensitivity analyses

Subgroup analyses indicated that *C*-indices were consistently higher for women compared to men and for MI compared to stroke for both derived scores in EPIC-Potsdam and EPIC-Heidelberg (SF4).

Calibration plots showed better calibration of the scores for women than men, with a more pronounced overestimation of risk for the higher decile groups of predicted risk in men (SF5).

Additional appraisal of CVD mortality discrimination resulted in higher *C*-indices for the derived scores than for SCORE in both cohorts (*C*-index EPIC-Heidelberg non-clinical: 0.774, 95%CI 0.525–0.960; clinical: 0.763, 0.513–0.954; SCORE: 0.740, 0.486–0.939). However, due to the limited number of fatal cases, estimates were imprecise.

## Discussion

We derived and externally validated a non-clinical risk score predicting 10-year CVD risk with superior or comparable performance to established clinical CVD risk scores. Additional clinical parameters only slightly improved discrimination. Our results suggest that estimation of 10-year CVD risk based on the selected and easily obtainable non-clinical CVD risk factor information is feasible without loss of predictive accuracy compared to clinical models.

Other external validations of the CMD Score showed acceptable to good discrimination in an Iranian (areas under the receiver operating characteristic curve (AUC): men 0.71, 95%CI 0.66–0.75; women 0.81, 0.76–0.85) and an Australian population (AUC: men 0.82, 0.77–0.86; women 0.88, 0.83–0.94) which is comparable or higher than in our samples^13,14^. Two meta-analyses, one based on 86 prospective studies, concluded that the PCE discriminates relatively well (*C*-index 0.723, 0.719–0.727) and reported a prediction interval (men 0.70, 0.60–0.79; women 0.74, 0.63–0.83) covering the observations from our study samples^15,16^. A pooled analysis of two other German population-based cohort studies showed a *C*-index (0.76, 0.73–0.79) comparable to our findings^17^. For the FRS including blood lipids, a meta-analysis of prospective studies reported a *C*-index of 0.719 (0.715–0.723), which is lower than in our cohorts^16^. For SCORE, the same meta-analysis suggested relatively good discrimination for all CVD events (*C*-index 0.719, 0.715–0.723) and better discrimination for fatal events only (*C*-index 0.758, 0.752–0.763)^16^. SCORE showed higher discriminatory ability in our samples when including all cases, while discrimination for fatal events was comparable. The PROCAM score for MI showed lower discrimination in other European validation studies than in our sample, with AUCs ranging from 0.55 to 0.74^18^.

The PCE endpoint definition (MI or coronary heart disease death, or fatal or non-fatal stroke) is largely comparable to our definition. While the PCE was well calibrated in a German sample after recalibration, our study still suggests slight overestimation of the recalibrated equation^17^. This could be related to deviations in the documented CVD incidence as a result of actual incidence differences in the studied populations and/or differences in the case identification and ascertainment procedure, potentially leading to systematically fewer or more identified cases. Additional inclusion of heart failure and angina in the FRS endpoint definition might explain the strong overestimation of risk detected in our samples.

Despite minor heterogeneity across individual validation studies potentially related to deviations in the population characteristics and covariate structure^19^, these findings indicate that the established clinical CVD models performed mostly comparable or better in EPIC-Potsdam and EPIC-Heidelberg compared to other studies. This suggests that underestimation of the performance in our samples is unlikely.

Several features of our approach are worth mentioning. Firstly, and most importantly, the developed non-clinical risk score extends individual risk prediction to prevention settings that are not covered by existing clinical risk scores without loss of predictive precision. These include self-assessment of individuals, health education campaigns, and step-wise screening procedures with a non-clinical stage. Secondly, the inclusion of selected GDRS parameters (age, waist circumference, smoking status, self-reported hypertension, consumption of whole grains, red meat, and coffee) in the non-clinical score allows simultaneous risk assessment of CVD and T2D with only a few additional parameters. Thirdly, our non-clinical score contains several lifestyle risk factors, modifiable and easily to be obtained, including dietary information. As effect sizes and directions of the modifiable predictors are in line with previous evidence (compare ST3), the score plausibly supports health behaviour recommendations, pointing out potential ways to reduce CVD risk, for example, by choice of a healthy diet or reducing waist circumference. Inclusion of behavioural over clinical parameters emphasises the role of primary lifestyle prevention rather than focussing on (medicinal) treatment of clinical parameters such as blood lipids or blood pressure, frequently used for CVD risk prediction, as potential consequences of adverse health behaviour. This is supported by our results showing that the investigated clinical parameters don’t provide much predictive information beyond our non-clinical predictors.

There are several strengths to our study. We based our analyses on physician-verified cases, reducing false-positive case assignment to a minimum. The application of the World Health Organization (WHO) Monitoring trends and determinants in cardiovascular disease (MONICA) criteria in the derivation cohort facilitates reproduction in other cohorts based on a standardised outcome definition. Harmonised data collection and procession methods between the EPIC centres in Potsdam and Heidelberg enabled us to fully rebuild the prediction model for external validation without regression or substitution of predictors that could be unavailable in other cohorts. Relevant sample sizes and case numbers in both cohorts (events per variable EPIC-Potsdam: non-clinical model 40.2, clinical model 136.8; events EPIC-Heidelberg n = 692) allowed the derivation of robust estimates, to perform sensitivity analyses, and to examine the performance in subgroups^20,21^.

However, there are some limitations. Firstly, due to the case-cohort design, the proportion of missingness was high for most biomarkers. However, it has been shown that multiple imputation is a valid approach to handle missing data for absolute risk estimations^22^. Secondly, we used the non-clinical score points as one predictor for the clinical score instead of individually modelling its risk factors. This approach may have diminished performance improvement. However, post-hoc re-estimation of the clinical model including the non-clinical risk factors individually showed that *C*-index increased only by 0.001, suggesting negligible loss of discriminatory ability. Thirdly, heterogeneous outcome definitions of the composite endpoint CVD may have hampered performance comparison with other risk scores, especially calibration. Finally, as we developed and validated our scores in German adults, generalisability to other populations with differences in case-mix and deviations in predictor and outcome assessment remains unclear.

To conclude, we developed and externally validated a non-clinical risk score predicting 10-year CVD risk based on shared predictors with a validated T2D risk score with comparable or superior performance to established clinical CVD risk scores. It can be used independently of physical examinations and includes a variety of modifiable risk factors supporting both, risk assessment and subsequent counselling for preventive lifestyle modifications, e.g., through an online calculator. The models will be implemented in the online tool of the GDRS (https://drs.dife.de/) and a paper questionnaire will be developed.

## Methods

### Study population

Analyses were based on the EPIC-Potsdam and EPIC-Heidelberg cohorts consisting of 27,548 and 25,540 participants recruited in the areas of Potsdam (age mainly 35–65 years, 60.4% female) and Heidelberg (age 35–66 years, 53.3% female). The data was collected from 1994 to 2012. Detailed information on recruitment and follow-up procedures is described elsewhere^23,24^. For baseline assessment, participants underwent physical examinations and blood sample drawing by trained medical personnel. Information on lifestyle, sociodemographic characteristics, and health status were documented with validated questionnaires and during face-to-face interviews. Participants were actively re-contacted every 2–3 years for follow-up information by sending questionnaires and phone calls if required. Additionally, passive follow-up sources like registry linkage or information of death certificates were used. Response rates ranged from 90 to 96% per follow-up round^23^.

In both cohorts, participants with prevalent CVD, non-verifiable, silent events, stroke cases with prior brain cancer, meninges, or leukaemia, and with missing follow-up information were excluded. Exclusively in EPIC-Potsdam, we excluded individuals with ‘possible’ events according to the WHO MONICA criteria. Exclusively in EPIC-Heidelberg, we excluded participants with events only indicated by a death certificate but without further sources suggesting an event. The analysis sample in EPIC-Potsdam contained 25,993 participants for the full follow-up, including 684 overall CVD cases (fatal n = 82), 383 myocardial infarctions (MI), and 315 stroke cases and after 10 years 584 overall CVD (fatal n = 70), 324 MI, and 269 stroke cases. Non-CVD death was documented for 2312 participants (8.9%) during the full follow-up and 847 participants (3.3%) within the first 10 years. The respective analysis sample in EPIC-Heidelberg contained 23,529 participants, including 692 overall CVD (fatal n = 87), 370 MI and 345 stroke cases after 10 years of follow-up (details: SF6). Non-CVD death was documented for 2596 participants (11.0%) during the full follow-up and 1074 participants (4.6%) during the first 10 years of follow-up. The studies were approved by the Ethical Committee of the State of Brandenburg and the Heidelberg University Hospital, Germany, and were carried out according to The Code of Ethics of the World Medical Association (Declaration of Helsinki). Participants gave written informed consent for participation.

### Assessment of predictors

Self-reported information on smoking, diet, prevalent hypertension and T2D, and medication was collected at baseline via questionnaires. Daily food consumption was assessed with self-administered semi-quantitative Food Frequency Questionnaires including photographs of portion sizes to estimate intake, summarised into food groups, and translated to portions per day as described elsewhere (overview of selected food groups and included dietary items: ST4)^25^. Waist circumference, systolic and diastolic blood pressure were measured by trained personnel at baseline examination (details: SN2). Biomarker measurements were performed in the established case-cohorts, consisting of a randomly drawn sample (subcohorts: Potsdam n = 2500; Heidelberg n = 2739) of participants who provided blood samples at baseline and incident cases of the according disease (case-cohorts: SF7, SN3, ST5; biomarker measurements: SN2)^26^. Family history of MI and stroke was collected at the 5th follow-up via questionnaires and summarised to parental and sibling history of CVD.

### Case ascertainment

Incident CVD was defined as all incident cases of non-fatal and fatal MI and stroke (International Statistical Classification of Diseases and Related Health Problems, Tenth revision (ICD-10) codes: I21 acute MI, I63.0–I63.9 ischemic stroke, I61.0–I61.9 intracerebral haemorrhage, I60.0–I60.9 subarachnoid haemorrhage, I64.0–I64.9 unspecified stroke). In both cohorts, events were systematically detected via self-report of a diagnosis, information of death certificates, and reports by local hospitals or treating physicians. If an event was indicated by the aforementioned sources, treating physicians were contacted for diagnosis verification, occurrence date, and diagnostic details. Only events with physician–verified diagnoses were considered as incident CVD cases. In EPIC-Potsdam, physician-verified cases were additionally ranked into ‘definite’, ‘probable’, and ‘possible’ events by two trained physicians based on the WHO MONICA criteria for MI and an adapted version for stroke (details: SN4).

### Statistical analyses

We applied multiple imputation by chained equations (m = 10) to handle missing values in predictor candidates and parameters needed to derive other scores for comparison (SN5)^27,28^.

Data of the EPIC-Potsdam cohort (follow-up time: median 11.35 years, IQR 1.38 years) was used for score derivation. We used the predictors of the GDRS in the first step and assessed their association with CVD using Cox proportional hazard regression in each imputed set separately^2,22,29^. Only parameters that were consistent in regards to effect size and direction with available meta-analyses or large-scale studies remained in the model. For the identification of CVD-specific predictor candidates, the literature was screened for established non-clinical and routinely available clinical CVD risk factors. To derive the non-clinical score, we considered candidates with regard to anthropometric measures, gender, CVD family history, self-reported prevalent diseases, medication, weight history, and dietary information as the main focus. The final selection of the predictors was based on the following criteria: performance improvement, assumed availability in physician-independent settings or routine care, consistency with previous evidence, and robustness of the association. Different predictor candidate combinations were added to the previously identified shared predictors from the GDRS to assess the independence and robustness of the associations. For the clinical extension, we used the score points of the non-clinical score as one predictor and subsequently added clinical candidates with regard to blood pressure measurements, blood pressure or lipid-lowering medication, blood lipid concentrations (total cholesterol, HDL cholesterol, and the respective ratio), and HbA_1c_. Predictor candidates meeting the previously defined criteria were included in the final scores.

Linearity assumptions of the risk associations were examined by deriving Martingale residuals and performing supremum tests for functional form^30^. The proportional hazards assumption was assessed by visual inspection of the Schoenfeld residuals.

Even though previous studies have commonly used Cox proportional hazards regression models for absolute risk predictions, including the PCE and FRS, non-CVD mortality is considered a competing risk event for the analysis of CVD endpoints. Despite a limited proportion of non-CVD mortality events in EPIC-Potsdam (3.3% during the first 10 years of follow-up), we additionally used Fine and Gray models accounting for competing risks, calculated absolute risks, assessed the model performance, and compared it to the performance of the Cox proportional hazards models^31,32^.

In a final step, we additionally considered squared terms and multiplicative interaction terms of the selected predictors with gender and age and, if statistically significantly associated with the outcome, added them to the model and re-evaluated the performance. To assess the potential benefit of modelling gender-stratified equations, we re-estimated the models in men and women separately and compared their performance.

*β* estimates of the final models were rounded, multiplied by 100 and the following equation including the subdistribution baseline survival *S*_*0*_ and mean values $\overline{{X}_{i}}$ of all participants was applied to calculate the absolute 10-year risks^29,33^:

$${\widehat{p}=1-{S}_{0}(t)}^{\mathrm{exp}(({\sum }_{i=1}^{p}{\beta }_{i } {X}_{i}-{\sum }_{i=1}^{p}{\beta }_{i }\overline{{X}_{i}})/100)}$$

We evaluated the performance of the generated scores in EPIC-Potsdam and for external validation in EPIC-Heidelberg censored at 10 years of follow-up and compared it to the performance of established CVD risk scores. Namely the non-clinical CMD risk score, the for Germany recalibrated PCE, two FRS including blood lipids or BMI, the ESC SCORE, and two PROCAM Scores predicting MI or stroke (calculation of scores: ST6)^3,10,17,33–35^. To quantify the discrimination of the scores, we calculated *C*-indices by using a bootstrap approach dividing each imputed set into 10 random subsets and adjusting for competing risks^36–38^. Calibration was assessed with calibration plots and expected-to-observed ratios. The calibration of the CMD score, SCORE, and both PROCAM scores was not evaluated due to differences in the predicted time frame or in the endpoint definitions (CVD mortality, MI, stroke). Potential changes in risk group assignment between the derived non-clinical and clinical score were assessed using the NRI with previously implemented risk groups (< 5%, ≥ 5%–< 7.5%, ≥ 7.5%–< 10%, ≥ 10%)^2^. Sensitivity and specificity were calculated based on the aforementioned risk cut-offs.

Sensitivity analyses were performed assessing the discrimination separately for men and women and for MI and stroke. For comparison with SCORE, we additionally calculated *C*-indices for fatal cases only.

Statistical analyses were performed with SAS (version 9.4).

## Supplementary Information

Supplementary Information 1.

Supplementary Information 2.

## Data Availability

The datasets analysed during the current study are not publicly available due to data protection regulations. In accordance with German Federal and State data protection regulations, epidemiological data analyses of EPIC-Potsdam may be initiated upon an informal enquiry addressed to the secretariat of the Human Study Center (Office.HSZ@dife.de). Each request will then have to pass a formal process of application and review by the respective Principal Investigator and a scientific board. The code for data analyses was written with SAS (version 9.4) and can be made available upon formal request.

## Abbreviations

95%CI: 95% Confidence interval
AUC: Area under the receiver operating characteristic curve
CMD: Chronic metabolic disease
CVD: Cardiovascular disease
EPIC: European Prospective Investigation into Cancer and Nutrition
FRS: Framingham CVD Risk Score
GDRS: German Diabetes Risk Score
ICD-10: International Statistical Classification of Diseases and Related Health Problems, Tenth revision
IQR: Interquartile range
MI: Myocardial infarction
MONICA: Monitoring trends and determinants in cardiovascular disease
NRI: Categorical Net-Reclassification-Improvement
PCE: Pooled Cohort Equations
SCORE: Systematic COronary Risk Evaluation
SF: Supplementary figure
sHR: Subdistribution hazard ratio
SN: Supplementary note
ST: Supplementary table
T2D: Type 2 diabetes
WHO: World Health Organization

## References

[1] Wilkins EW, Wilson L, Wickramasinghe K, Bhatnagar P, Leal J, Luengo-Fernandez R, Burns R, Rayner M, Townsend N (2017). European Cardiovascular Disease Statistics 2017.

[2] Goff DC, Lloyd-Jones DM, Bennett G, Coady S, D’Agostino RB, Gibbons R (2014). 2013 ACC/AHA Guideline on the Assessment of Cardiovascular Risk: A report of the American College of Cardiology/American Heart Association Task Force on Practice Guidelines. J. Am. Coll. Cardiol..

[3] Conroy RM, Pyorala K, Fitzgerald AP, Sans S, Menotti A, De Backer G (2003). Estimation of ten-year risk of fatal cardiovascular disease in Europe: The SCORE project. Eur. Heart J..

[4] Arnett DK, Blumenthal RS, Albert MA, Buroker AB, Goldberger ZD, Hahn EJ (2019). 2019 ACC/AHA Guideline on the Primary Prevention of Cardiovascular Disease: A report of the American College of Cardiology/American Heart Association Task Force on Clinical Practice Guidelines. J. Am. Coll. Cardiol..

[5] Piepoli MF, Hoes AW, Agewall S, Albus C, Brotons C, Catapano AL (2016). 2016 European Guidelines on cardiovascular disease prevention in clinical practiceThe Sixth Joint Task Force of the European Society of Cardiology and Other Societies on Cardiovascular Disease Prevention in Clinical Practice (constituted by representatives of 10 societies and by invited experts) developed with the special contribution of the European Association for Cardiovascular Prevention; Rehabilitation (EACPR). Eur. Heart J..

[6] Aslibekyan S, Campos H, Loucks EB, Linkletter CD, Ordovas JM, Baylin A (2011). Development of a cardiovascular risk score for use in low- and middle-income countries. J. Nutr..

[7] McGorrian C, Yusuf S, Islam S, Jung H, Rangarajan S, Avezum A (2010). Estimating modifiable coronary heart disease risk in multiple regions of the world: The INTERHEART Modifiable Risk Score. Eur. Heart J..

[8] Qiao Q, Gao W, Laatikainen T, Vartiainen E (2011). Layperson-oriented vs. clinical-based models for prediction of incidence of ischemic stroke: National FINRISK study. Int. J. Stroke..

[9] Chiuve SE, Cook NR, Shay CM, Rexrode KM, Albert CM, Manson JE (2014). Lifestyle-based prediction model for the prevention of CVD: The Healthy Heart Score. J. Am. Heart Assoc..

[10] Alssema M, Newson RS, Bakker SJ, Stehouwer CD, Heymans MW, Nijpels G (2012). One risk assessment tool for cardiovascular disease, type 2 diabetes, and chronic kidney disease. Diabetes Care.

[11] Schulze MB, Martinez-Gonzalez MA, Fung TT, Lichtenstein AH, Forouhi NG (2018). Food based dietary patterns and chronic disease prevention. BMJ.

[12] Muhlenbruch K, Ludwig T, Jeppesen C, Joost HG, Rathmann W, Meisinger C (2014). Update of the German Diabetes Risk Score and external validation in the German MONICA/KORA study. Diabetes Res. Clin. Pract..

[13] Asgari S, Moosaie F, Khalili D, Azizi F, Hadaegh F (2020). External validation of the European risk assessment tool for chronic cardio-metabolic disorders in a Middle Eastern population. J. Transl. Med..

[14] Rauh SP, Rutters F, van der Heijden AAWA, Luimes T, Alssema M, Heymans MW (2018). External validation of a tool predicting 7-year risk of developing cardiovascular disease, type 2 diabetes or chronic kidney disease. J. Gen. Intern. Med..

[15] Damen JA, Pajouheshnia R, Heus P, Moons KGM, Reitsma JB, Scholten R (2019). Performance of the Framingham risk models and pooled cohort equations for predicting 10-year risk of cardiovascular disease: A systematic review and meta-analysis. BMC Med..

[16] Pennells L, Kaptoge S, Wood A, Sweeting M, Zhao X, White I (2019). Equalization of four cardiovascular risk algorithms after systematic recalibration: Individual-participant meta-analysis of 86 prospective studies. Eur. Heart J..

[17] de Las Heras Gala T, Geisel MH, Peters A, Thorand B, Baumert J, Lehmann N (2016). Recalibration of the ACC/AHA risk score in two population-based German cohorts. PLoS ONE.

[18] Siontis GCM, Tzoulaki I, Siontis KC, Ioannidis JPA (2012). Comparisons of established risk prediction models for cardiovascular disease: Systematic review. BMJ Br. Med. J..

[19] Damen J, Debray TPA, Pajouheshnia R, Reitsma JB, Scholten R, Moons KGM (2019). Empirical evidence of the impact of study characteristics on the performance of prediction models: A meta-epidemiological study. BMJ Open.

[20] Riley RD, Ensor J, Snell KIE, Harrell FE, Martin GP, Reitsma JB (2020). Calculating the sample size required for developing a clinical prediction model. BMJ.

[21] Vergouwe Y, Steyerberg EW, Eijkemans MJ, Habbema JD (2005). Substantial effective sample sizes were required for external validation studies of predictive logistic regression models. J. Clin. Epidemiol..

[22] Muhlenbruch K, Kuxhaus O, di Giuseppe R, Boeing H, Weikert C, Schulze MB (2017). Multiple imputation was a valid approach to estimate absolute risk from a prediction model based on case-cohort data. J. Clin. Epidemiol..

[23] Bergmann MM, Bussas U, Boeing H (1999). Follow-up procedures in EPIC-Germany—data quality aspects European Prospective Investigation into Cancer and Nutrition. Ann. Nutr. Metab..

[24] Boeing H, Korfmann A, Bergmann MM (1999). Recruitment procedures of EPIC-Germany. European investigation into cancer and nutrition. Ann. Nutr. Metab..

[25] Schulze MB, Hoffmann K, Kroke A, Boeing H (2001). Dietary patterns and their association with food and nutrient intake in the European Prospective Investigation into Cancer and Nutrition (EPIC)-Potsdam study. Br. J. Nutr..

[26] Boeing H, Wahrendorf J, Becker N (1999). EPIC-Germany—A source for studies into diet and risk of chronic diseases. European Investigation into Cancer and Nutrition. Ann. Nutr. Metab..

[27] Raghunathan TE, Lepkowski JM, Van Hoewyk J, Solenberger P (2001). A multivariate technique for multiply imputing missing values using a sequence of regression models. Surv. Methodol..

[28] van Buuren S (2007). Multiple imputation of discrete and continuous data by fully conditional specification. Stat. Methods Med. Res..

[29] Cox DR (1972). Regression models and life-tables. J. R. Stat. Soc. Ser. B Methodol..

[30] Lin DY, Wei LJ, Ying Z (1993). Checking the Cox model with cumulative sums of martingale-based residuals. Biometrika.

[31] Fine JP, Gray RJ (1999). A proportional hazards model for the subdistribution of a competing risk. J. Am. Stat. Assoc..

[32] Kohl M, Plischke M, Leffondré K, Heinze G (2015). PSHREG: A SAS macro for proportional and nonproportional subdistribution hazards regression. Comput. Methods Programs Biomed..

[33] D'Agostino RB, Vasan RS, Pencina MJ, Wolf PA, Cobain M, Massaro JM (2008). General cardiovascular risk profile for use in primary care: The Framingham Heart Study. Circulation.

[34] Assmann G, Cullen P, Schulte H (2002). Simple scoring scheme for calculating the risk of acute coronary events based on the 10-year follow-up of the prospective cardiovascular Munster (PROCAM) study. Circulation.

[35] Assmann G, Schulte H, Cullen P, Seedorf U (2007). Assessing risk of myocardial infarction and stroke: New data from the Prospective Cardiovascular Munster (PROCAM) study. Eur. J. Clin. Investig..

[36] Pencina MJ, D'Agostino RB (2004). Overall C as a measure of discrimination in survival analysis: Model specific population value and confidence interval estimation. Stat. MED..

[37] Cook N. C-statistics for survival data: SAS Macro %predc. http://ncook.bwh.harvard.edu/sas-macros.html (Accessed 15 Aug 2019).

[38] Wolbers M, Koller MT, Witteman JCM, Steyerberg EW (2009). Prognostic models with competing risks: Methods and application to coronary risk prediction. Epidemiology.

