# Supplementary material for: A newly developed and externally validated non-clinical score accurately predicts 10-year cardiovascular disease risk in the general adult population
Source: Sci Rep. 2021 Oct 4;11:19609. doi: 10.1038/s41598-021-99103-4 (PMC8490374; doi:10.1038/s41598-021-99103-4)
Supplement: Supplementary file 1 — Supplementary Information 1. [file 41598_2021_99103_MOESM1_ESM.pdf]

Supplementary Material for Manuscript:

A newly developed and externally validated non-clinical score accurately predicts 10-year cardiovascular disease risk in the general adult population

Catarina Schiborn, PhD [1, 2], Tilman Kühn, PhD [3, 4], Kristin Mühlenbruch, PhD [1, 2], Olga Kuxhaus, Diploma[1], Cornelia Weikert, MD [5, 6], Andreas Fritsche, PhD [2, 7, 8], Rudolf Kaaks, PhD [3], Matthias B. Schulze, DrPH [1, 2, 9]

[1] Department of Molecular Epidemiology, German Institute of Human Nutrition Potsdam-Rehbruecke (DIfE), Nuthetal, Germany.

[2] German Center for Diabetes Research (DZD), Munich Neuherberg, Germany.

[3] Division of Cancer Epidemiology, German Cancer Research Center (DKFZ), Heidelberg, Germany.

[4] Institute for Global Food Security, Queen's University Belfast, Belfast, UK

[5] Department of Food Safety, German Federal Institute for Risk Assessment, Berlin, Germany.

[6] Institute for Social Medicine, Epidemiology and Health Economics, Charité University Medical Center, Berlin, Germany.

[7] Institute for Diabetes Research and Metabolic Diseases of the Helmholtz Center Munich at the University of Tübingen, Tübingen, Germany.

[8] Division of Endocrinology, Diabetology, Vascular Disease, Nephrology and Clinical Chemistry, Department of Internal Medicine, University of Tübingen, Tübingen, Germany.

[9] Institute of Nutritional Science, University of Potsdam, Nuthetal, Germany

Corresponding author:

Catarina Schiborn

Tel: +49 33200 88 2526

Fax: +49 33200 88 2437

Mail: catarina.schiborn@dife.de

Contents:

Supplementary Notes

Supplementary Note 1 Example of absolute risk calculation with the derived non-clinical and clinical score 3

Supplementary Note 2 Measurement of blood pressure and biomarkers 3

Supplementary Note 3 Information on embedded case-cohorts 3

Supplementary Note 4 Further information on case ascertainment 4

Supplementary Note 5 Information on imputation procedure and summary methods 4

Supplementary Figures

Supplementary Figure 1 Restricted cubic spline for the covariate 'CVD points' exemplarily derived in the first imputed set to assess linearity of risk association..............5

Supplementary Figure 2 Calibration of the non-clinical and clinical CVD-scores based on (A) Cox proportional hazards regression and (B) Fine and Gray competing risk models..............6

Supplementary Figure 3 Overall performance for the non-clinical and clinical score with (A) additional significant interactions and significant squared terms and (B) the re-estimated models, derived in women and men separately in EPIC-Potsdam. 7

Supplementary Figure 4 Discrimination of the derived non-clinical and clinical scores in EPIC-Potsdam and EPIC-Heidelberg, depicted as *C*-indices and 95% confidence intervals (95% CI) in men and women separately or for myocardial infarction (MI) and stroke respectively. 8

Supplementary Figure 5 Calibration plots stratified by gender in the EPIC-Potsdam and EPIC-Heidelberg cohorts by decile of predicted risk with the according 95% confidence interval (95%CI) for the (A) non-clinical and (B) clinical score. 9

Supplementary Figure 6 Exclusion flow chart depicting number of excluded individuals and reasons for exclusion in the EPIC-Potsdam and EPIC-Heidelberg cohorts 10

Supplementary Figure 7 Scheme of the subcohort and case cohorts embedded in the overall cohort 11

Supplementary Tables

Supplementary Table 1 Comparison of the distribution of parameters included in the imputation models for the EPIC-Potsdam and EPIC-Heidelberg cohorts before and after imputation.. 12

Supplementary Table 2 Sensitivity, specificity, positive predictive value (PPV), and negative predictive value (NPV) of the derived non-clinical and clinical score in EPIC-Potsdam and EPIC-Heidelberg. 20

Supplementary Table 3 Risk association of the predictors included in the developed scores with cardiovascular events in other meta-analyses or large-scale studies 21

Supplementary Table 4 Selected food groups derived from the Food Frequency Questionnaire (FFQ) in EPIC-Potsdam and EPIC-Heidelberg and the according summarised food items. 24

Supplementary Table 5 Number of participants of the overall EPIC-Potsdam and EPIC-Heidelberg cohorts, the randomly drawn subcohort, cardiovascular disease (CVD), type 2 diabetes (T2D), and transient ischemic attack (TIA) cases respectively up to the time point of sampling and the according overlap with the subcohort. 24

Supplementary Table 6 Parameters and according equations used for calculation of absolute risks with external scores for comparison. 25

Supplementary Note 1 Example of absolute risk calculation with the derived non-clinical and clinical score

Non-clinical Score:

Woman, 56 years old, waist circumference 80 cm, former heavy smoker, has self-reported hypertension, one parent with CVD, consumes one portion whole grain and coffee, 0.5 portions of red meat and 2 portions of plant oil per day

Calculate points

$$points_{\ nonclin} = \sum_{i=1}^{p} \beta_i X_i = 7.6 * 56 + 1*80 + 9 + 48 + 14 - 11 * 1 - 3 * 1 + 36 * 0.5 - 13 * 2 = 581.6$$

Derive absolute risk

$$\hat{p}_{example\ nonclin} = 1 - 0.98614^{\exp\left(\frac{581.6-517.7665}{100}\right)} = 2.61\ \%$$

The probability to have a CVD in the following 10 years is 2.61%.

Clinical extension:

Same woman with a systolic blood pressure of 130 mmHg and diastolic of 80 mmHg, total cholesterol of 220mg/dl and HDL cholesterol of 40 mg/dl

Calculate points

$$points_{\ clin} = \sum_{i=1}^{p} \beta_i X_i = 0.89 * 581.6 + 0.57*130 + 1.25 * 80 + 0.38 * 220 - 0.48 * 40 = 756{,}124$$

Derive absolute risk

$$\hat{p}_{example\ clin} = 1 - 0.98683^{\exp\left(\frac{756{,}124-691.066}{100}\right)} = 2.51\ \%$$

The probability to have a CVD in the following 10 years is 2.51%.

Supplementary Note 2 Measurement of blood pressure and biomarkers

Blood pressure was assessed with an automated oscillometric device (BOSO Oscillomat®, Jungingen, Germany) after 5 minutes resting time by performing three readings on the right arm with 2-minute intervals in between while sitting [1]. Results of the second and third reading were averaged.

Blood samples of 30ml per participant were taken at baseline examination under standardised conditions. Of those, 20ml were filled into monovettes with citrate as anticoagulant. Blood was separated into serum, plasma, buffy coat, and erythrocytes and was aliquoted into straws of 0.5 ml each and stored in liquid nitrogen (–196°C). In EPIC-Potsdam, baseline total cholesterol, HDL cholesterol, and triglyceride concentrations were measured in plasma and HbA1c in serum samples at the University Clinic Tübingen using the automatic ADVIA® analyzer (Siemens Medical Solutions, Erlangen, Germany) following the manufacturer's instructions. To account for plasma dilution with citrate, biomarker values were multiplied by 1.16 in women and by 1.17 in men. In EPIC-Heidelberg, total cholesterol, HDL cholesterol, and triglyceride concentrations were measured in plasma and HbA1c from erythrocytes in serum samples using the Roche Cobas 6000 analytical system for clinical chemistry following the manufacturer's instructions in the Scandinavian Health Ltd. laboratories (Etten-Leur, Netherlands). The proportion of fasted participants at blood sample draw was 28% in EPIC-Potsdam and 7.9% in EPIC-Heidelberg.

Supplementary Note 3 Information on embedded case-cohorts

Measurements of biomarkers were available in the subcohorts in EPIC-Potsdam and EPIC-Heidelberg that were randomly drawn from participants who provided blood samples at baseline examination (Potsdam: 95.7%; Heidelberg: 95.8%). In EPIC-Potsdam, biomarkers were additionally measured in all incident type 2 diabetes (T2D) cases identified until August 31st 2005 as part of the T2D case-cohorts, all incident CVD as myocardial infarction (MI) and stroke cases as part of the CVD case-cohort and all incident transient ischemic attack (TIA) cases identified until November 30th 2006. In EPIC-Heidelberg, biomarkers were measured in the subcohort and all incident CVD (MI and stroke) cases identified until December 31st 2006 as part of the CVD case cohort. A scheme of the case-cohort structure is depicted in the Supplementary Figure 2.

Supplementary Note 4 Further information on case ascertainment

Classification of the events according to the WHO MONICA criteria was conducted by two trained physicians based on verification sheets that were sent out to the treating physicians. The verification sheet for MI requests among others information on date of diagnosis, presence of coronary heart disease, details on the diagnosis regarding symptoms, ECG and heart enzymes. The verification sheet for stroke requests among others information on date of diagnosis, according ICD-10 code and details on the diagnosis regarding anamnesis, clinical symptoms, CT/MRI, angiogram, lumbar puncture, echocardiogram, Doppler ultrasonography, electrocardiogram and fatality of the event. For stroke, apart from the WHO MONICA criteria, the described imaging techniques were taken into account to classify events into ‘definite’, ‘probable’ and ‘possible’ cases in EPIC-Potsdam. If a MI and a stroke occurred on the same day, the case was classified as a MI event. Events that were not identified and diagnosed within 48 hours after occurrence were classified as silent events and according to the WHO MONICA criteria excluded from the analyses as no exact date of occurrence could be defined.

Supplementary Note 5 Information on imputation procedure and summary methods

Parameters from the following areas were eligible for the imputation model as explanatory variables: diet, physical activity, education, medication, prevalent cardiometabolic diseases, alcohol consumption, smoking, anthropometry, weight history, family history of diabetes and measured biomarkers. They were included in the model if they met at least one of the following criteria: assumed to explain the missingness pattern, assumed to contain information on values of the missing parameters, correlated $r \geq 0.2$ or $r \leq -0.2$ with the missing parameters. We furthermore included all analysed outcome variables in the models. As recommended by White, instead of the follow up time, we calculated the according Nelson Aalen estimate and used it in the imputation [2]. Continuous variables were transformed by BoxCox transformation to fit normal distribution before imputation. To select the appropriate lambda we used PROC Transreg. If parameters contained 0 values, we added a constant of 1 before assessment of suitable lambda [3]. Continuous parameters were imputed by performing predictive mean matching (k=5) [4]. Binary parameters were imputed following a logistic distribution. Nominally and ordinal scaled parameters were imputed by using the discrim statement.

To summarise the β estimates derived by Cox proportional hazards regression and Fine and Gray competing risk models in the imputed sets, we used Rubin’s rules [5]. Baseline survivals S_0 were calculated per imputed set and summarised by applying complementary log-log transformation [6]. Mean values of all participants were calculated in each imputed set and summarised by Rubin’s rules. The performance measures derived in each imputed set were summarised by calculating the median of the imputed sets and the respective 95% confidence interval (95%CI).

Model assumptions of the Cox proportional hazards regression were tested in each imputed set separately and violations in more than 5 sets were considered as overall violated.

Figures

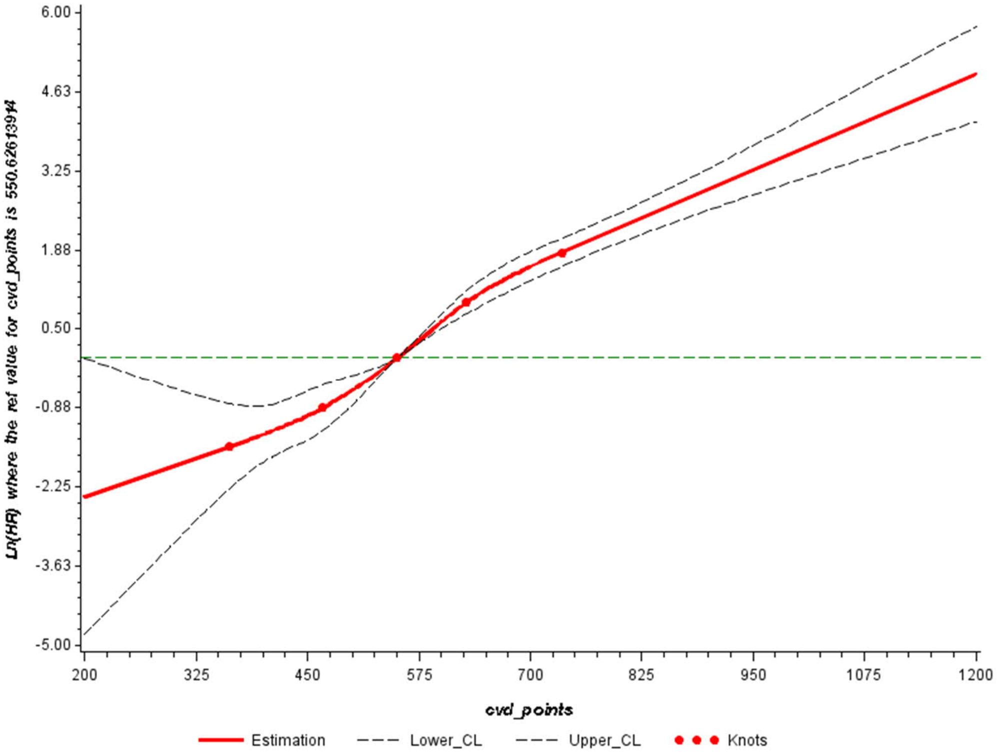

Supplementary Figure 1 Restricted cubic spline for the covariate 'CVD points' exemplarily derived in the first imputed set to assess linearity of risk association; knots p5, p25, p50, p75, p95; adjusted for: Systolic blood pressure [mmHg], diastolic blood pressure [mmHg], total cholesterol [mg/dl], HDL cholesterol [mg/dl]

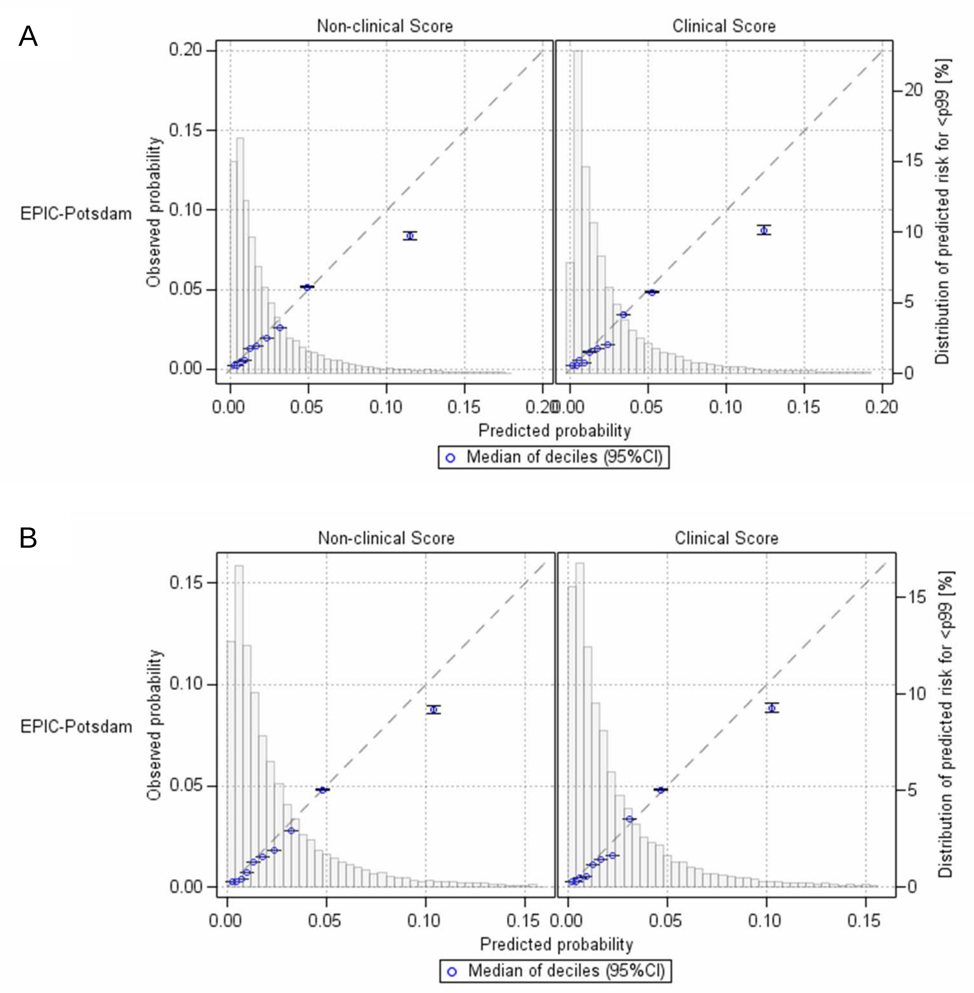

Supplementary Figure 2 Calibration of the non-clinical and clinical CVD-scores based on (A) Cox proportional hazards regression and (B) Fine and Gray competing risk models. Calibration is depicted as calibration plot by decile of predicted risk. Distribution of predicted risk up to the 99th percentile (p) is depicted in the background.

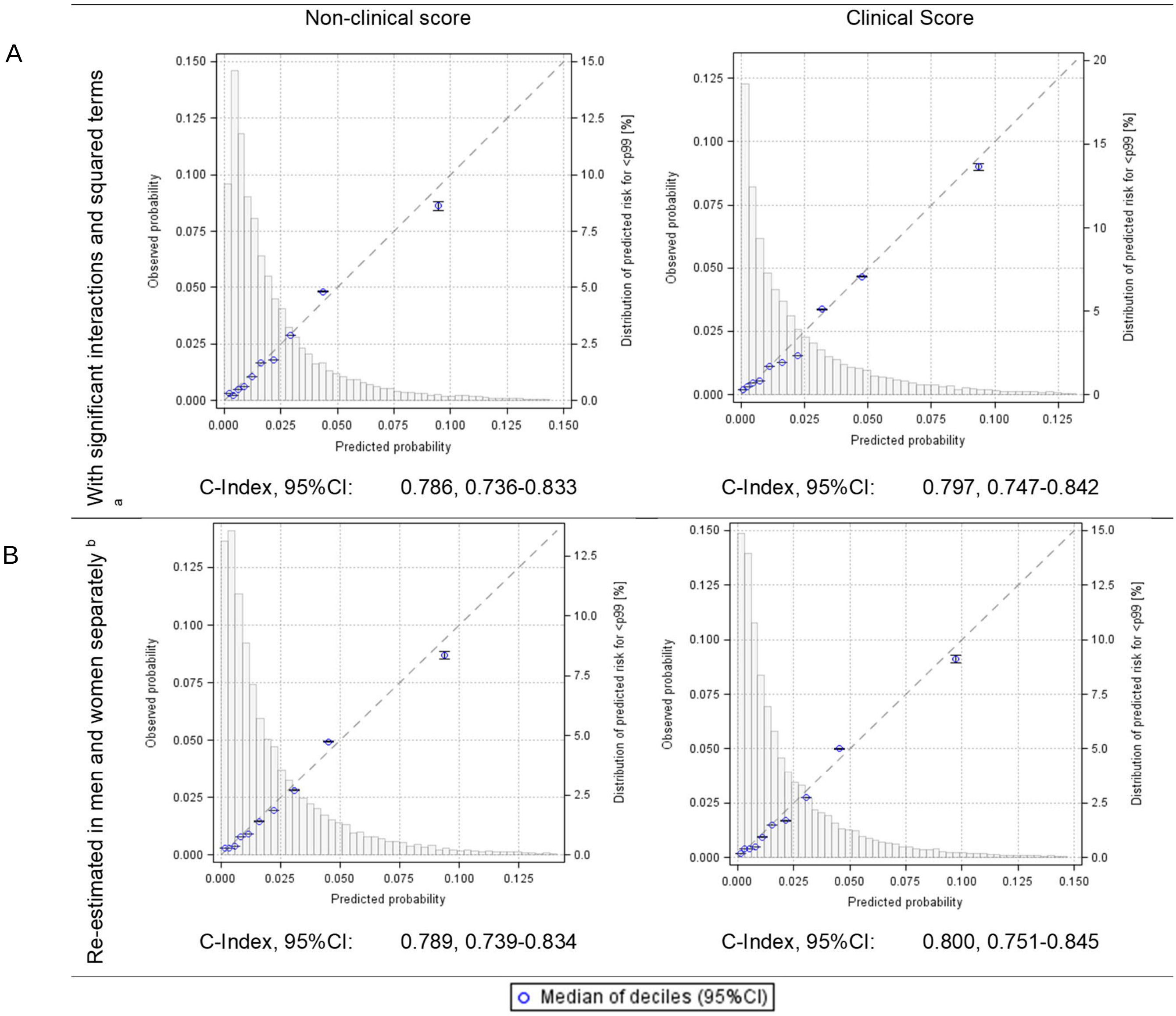

Supplementary Figure 3 Overall performance for the non-clinical and clinical score with (A) additional significant interactions and significant squared terms and (B) the re-estimated models, derived in women and men separately in EPIC-Potsdam; Calibration is depicted as calibration plot by decile of predicted risk. Distribution of predicted risk up to the 99th percentile (p) is depicted in the background. Discrimination is shown as *C*-indices and the 95% confidence interval (95%CI).

[a] a significant interaction was detected for age* plant oil consumption; significant squared terms were detected for coffee consumption, non-clinical CVD points, and HDL cholesterol

[b] Re-estimated models for men and women (sex specific estimates of β coefficients, baseline and mean risk) excluding gender as a predictor

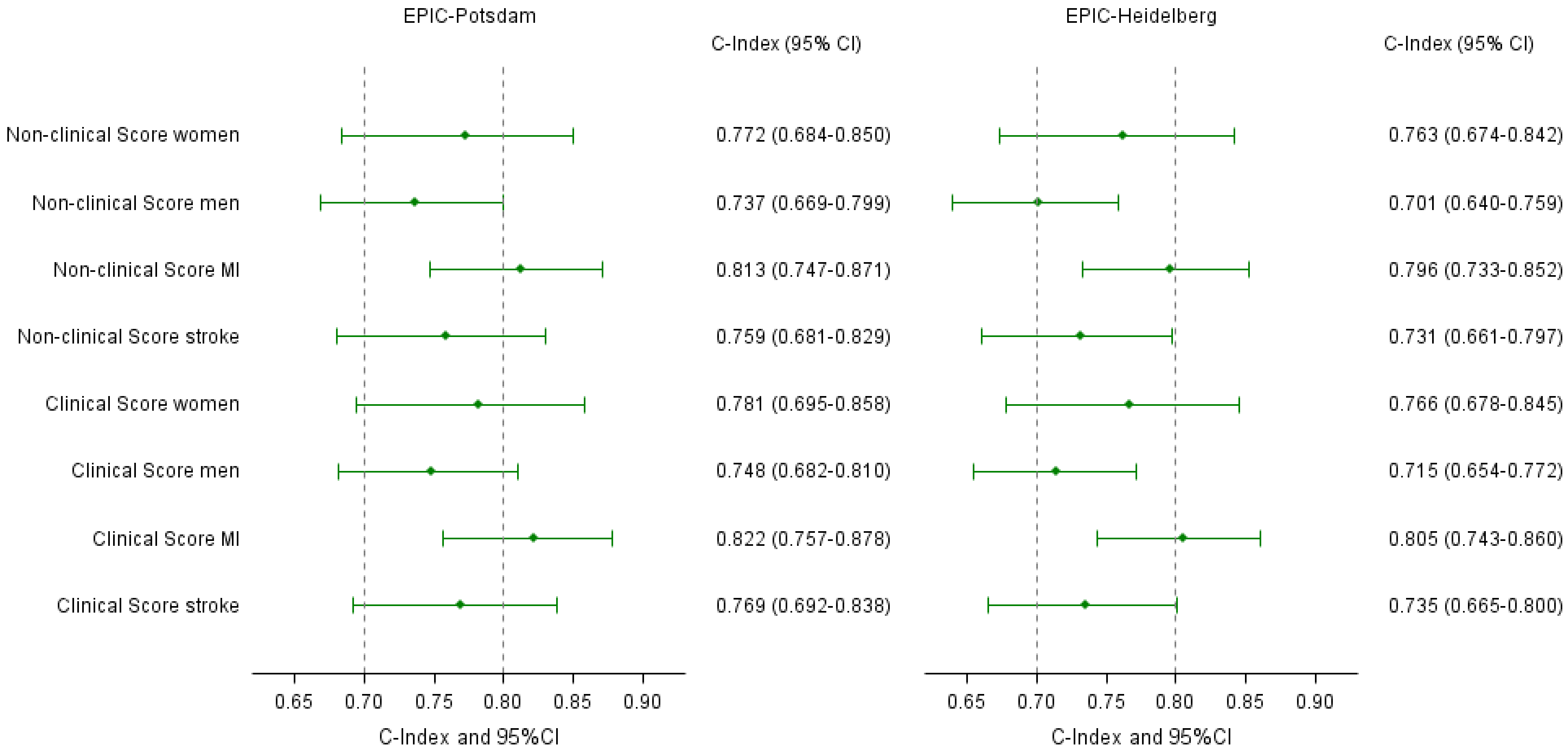

Supplementary Figure 4 Discrimination of the derived non-clinical and clinical scores in EPIC-Potsdam and EPIC-Heidelberg, depicted as *C*-indices and 95% confidence intervals (95% CI) in men and women separately or for myocardial infarction (MI) and stroke respectively. EPIC, European Prospective Investigation into Cancer and Nutrition.

A

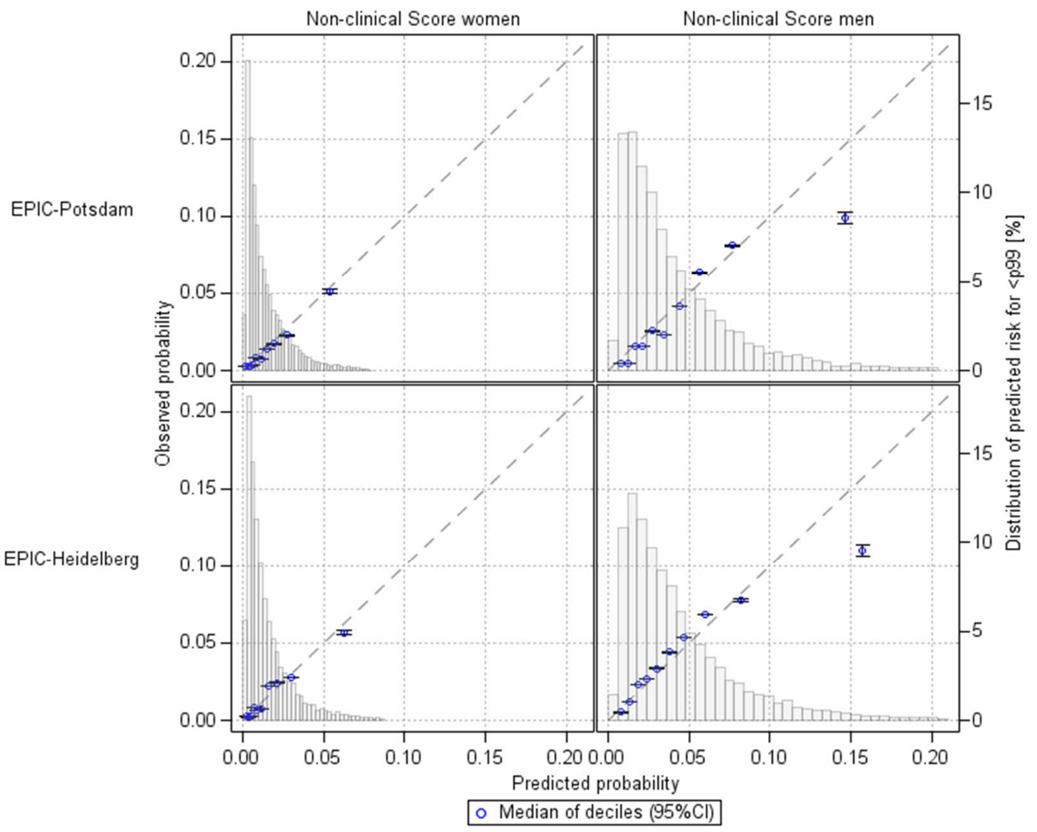

B

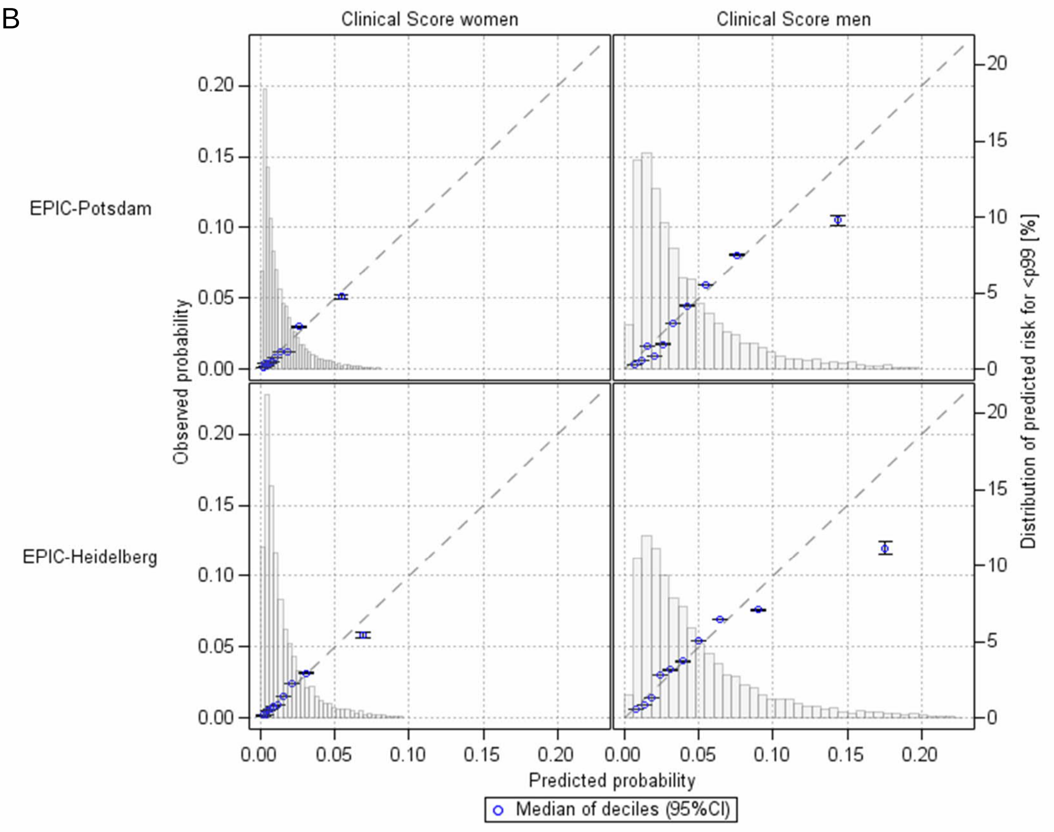

Supplementary Figure 5 Calibration plots stratified by gender in the EPIC-Potsdam and EPIC-Heidelberg cohorts by decile of predicted risk with the according 95% confidence interval (95%CI) for the (A) non-clinical and (B) clinical score. Distribution of predicted risk up to the 99th percentile (p) is depicted in the background.

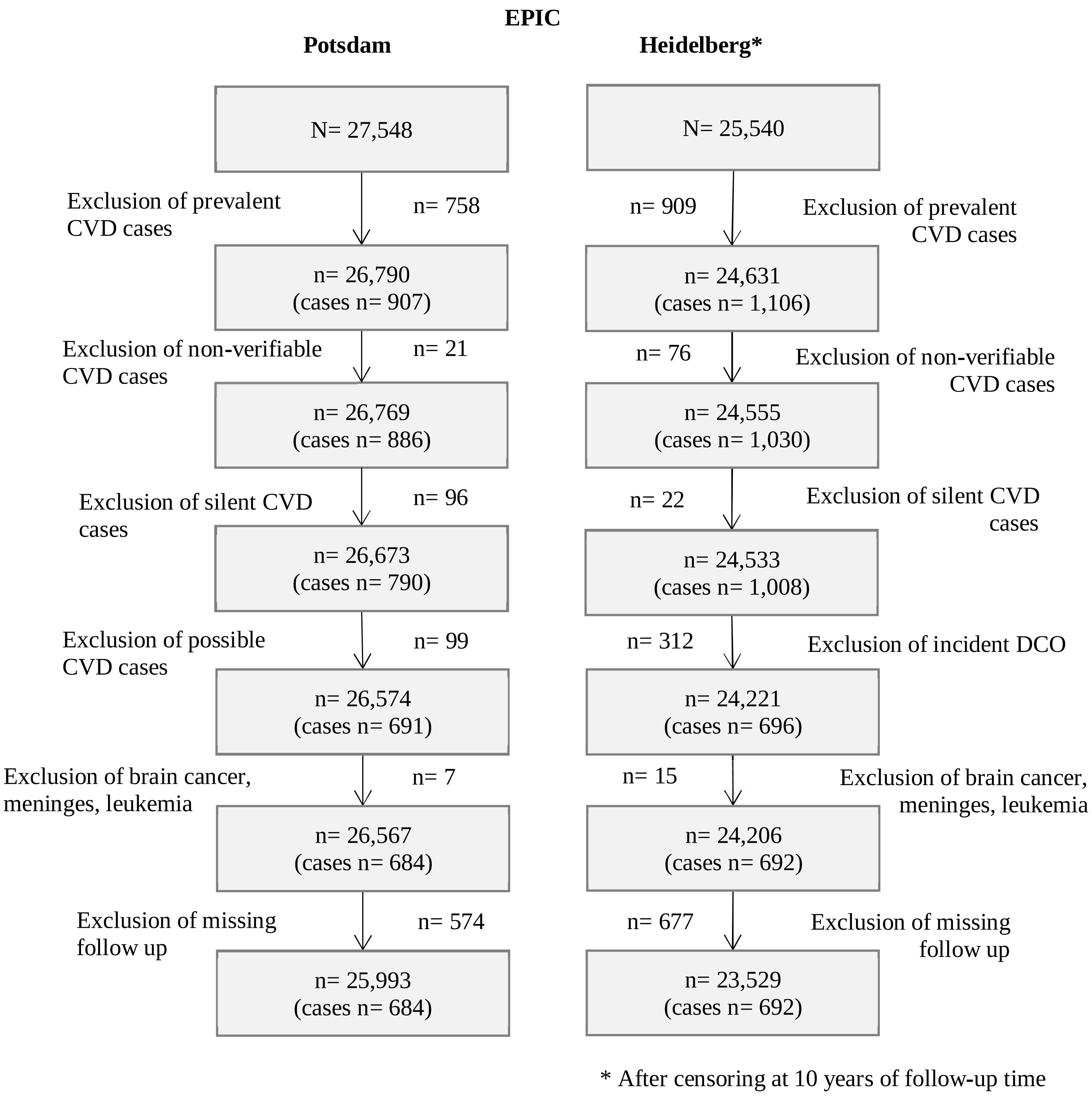

Supplementary Figure 6 Exclusion flow chart depicting number of excluded individuals and reasons for exclusion in the EPIC-Potsdam and EPIC-Heidelberg cohorts. EPIC-Heidelberg data was censored at 10 years of follow-up. CVD, cardiovascular disease; DCO, death certificate only

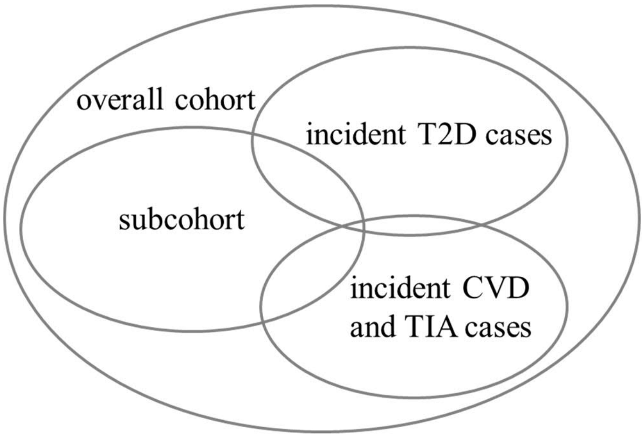

Supplementary Figure 7 Scheme of the subcohort and case cohorts embedded in the overall cohort. T2D, type 2 diabetes. CVD, cardiovascular disease. TIA, transient ischemic attack.

Tables

Supplementary Table 1 Comparison of the distribution of parameters included in the imputation models for the EPIC-Potsdam and EPIC-Heidelberg cohorts before and after imputation. Values depicted as median and interquartile range or proportion. Median and IQR or proportion after imputation were calculated across all 10 imputed sets.

Parameters included in imputation procedure	Missing values			Median (IQR) or proportion			
	Number	Proportion (%)	Lambda[a]	Before imputation		After imputation	
**EPIC-Potsdam (n=27,548)**							
Case diabetes (yes)	0	0.00		5.70 %		5.70 %	
Case MI (yes)	0	0.00		1.43 %		1.43 %	
Case stroke (yes)	0	0.00		1.22 %		1.22 %	
Case CVD (yes)	0	0.00		2.48 %		2.48 %	
Censoring date Follow-up 5	0	0.00					
Gender (female)	0	0.00		60.42 %		60.42 %	
Age at recruitment	0	0.00		50.79	(15.80)	50.79	(15.80)
Hypertension medications (yes)	0	0.00		19.79 %		19.79 %	
Nelson-Aalen estimate for MI follow-up	0	0.00		0.0154176	(0.0027825)	0.0154176	(0.0027825)
Nelson-Aalen estimate for stroke follow-up	0	0.00		0.0137520	(0.0022047)	0.0137520	(0.0022047)
Nelson-Aalen estimate for T2D follow up	0	0.00		0.0659497	(0.0116755)	0.0659497	(0.0116755)
Nelson-Aalen estimate for CVD follow-up	0	0.00		0.0276959	(0.0046441)	0.0276959	(0.0046441)
Subcohort member (yes)	0	0.00		9.08 %		9.08 %	
CVD case (case-cohort) (yes)	0	0.00		1.84 %		1.84 %	
TIA case (case-cohort) (yes)	0	0.00		0.87 %		0.87 %	
T2D case (case-cohort) (yes)	0	0.00		2.98 %		2.98 %	
Basic metabolic rate	0	0.00		6.17	(1.63)	6.17	(1.63)
Min. 1 follow-up questionnaire (yes)	0	0.00		97.03 %		97.03 %	
Hyperlipidaemia medication (yes)	0	0.00		5.42 %		5.42 %	
Diabetes medications (yes)	0	0.00		2.71 %		2.71 %	
Ever hyperlipidaemia or drugs for hyperlipidaemia (yes)	0	0.00		27.58 %		27.58 %	
Highest school level - collage of higher education, university (yes)	0	0.00		36.47 %		36.47 %	
Alcohol intake (<=6 g/day) (yes)	0	0.00		39.73 %		39.73 %	
Alcohol intake (>24-60 g/day) (yes)	0	0.00		14.60 %		14.60 %	

[continued on next page]

	Missing values			Median (IQR) or proportion	
**Parameters included in imputation procedure**	**Number**	**Proportion (%)**	**Lambda[a]**	**Before imputation**	**After imputation**
**EPIC-Potsdam (n=27,548)**					
Case classification according to WHO MONICA criteria	0	0.00			
MI					
definite				87.17 %	87.17 %
probable				6.18 %	6.18 %
possible				6.65 %	6.65 %
Stroke	0	0.00			
definite				78.33 %	78.33 %
probable				3.81 %	3.81 %
possible				17.86 %	17.86 %
Hypertension, self-reported at baseline (yes)	11	0.04		32.88 %	32.88 %
Hyperlipidaemia, self-reported at baseline	11	0.04			
yes				27.37 %	27.37 %
no				72.08 %	72.07 %
don't know				0.56 %	0.56 %
Sport, biking, gardening [h/week]	11	0.04	0.25	4.5 (6.5)	4.5 (6.5)
Whole-grain bread + cereals (50g portion/day)	17	0.06	-0.75	0.60 (1.32)	0.59 (1.33)
Coffee (150g portion/day)	17	0.06	0.25	2.00 (2.58)	2.00 (2.58)
Red meat (150g portion/day)	17	0.06	-2.25	0.24 (0.21)	0.24 (0.21)
Smoking status	17	0.06			
Never				47.01 %	47.01 %
Former [<20 units/d]				22.95 %	22.94 %
Former [≥20 units/d]				9.43 %	9.42 %
Current [<20 units/d]				14.81 %	14.81 %
Current [≥20 units/d]				5.80 %	5.81 %
Alcohol intake from beverages [g/day]	17	0.06	0.25	7.79 (16.24)	7.8 (16.25)
Other bread [g/d]	17	0.06	0.5	119.74 (103.56)	119.71 (103.59)
Fresh fruit [g/d]	17	0.06	0.25	100.33 (106.58)	100.33 (106.58)
Canned fruit [g/d]	17	0.06	0	9.86 (15.12)	9.86 (15.12)
Raw vegetables [g/d]	17	0.06	0.25	46.51 (41.06)	46.52 (41.07)
Cooked vegetables [g/d]	17	0.06	0.25	25.34 (20.32)	25.34 (20.32)
Legumes [g/d]	17	0.06	0	17.17 (23.22)	17.17 (23.21)
Fried potatoes [g/d]	17	0.06	0.25	9.76 (14.01)	9.76 (14.01)
Nuts [g/d]	17	0.06	-0.75	0.82 (3.70)	0.82 (3.70)

[continued on next page]

Parameters included in imputation procedure	Missing values: Number	Proportion (%)	Lambda[a]	Median (IQR) or proportion: Before imputation		After imputation	
**EPIC-Potsdam (n=27,548)**							
Low-fat dairy products [g/d]	17	0.06	0	22.60	(131.29)	22.60	(131.29)
High-fat dairy products [g/d]	17	0.06	1	50.96	(126.05)	50.88	(126.08)
Low-fat cheese [g/d]	17	0.06	1	0.00	(5.68)	0.00	(5.69)
High-fat cheese [g/d]	17	0.06	0.25	24.44	(27.72)	24.44	(27.72)
Fruit juice [g/d]	17	0.06	0.25	124.14	(221.49)	124.10	(221.40)
Low-energy soft drinks [g/d]	17	0.06	-2	0.00	(0.00)	0.00	(0.00)
High-energy soft drinks [g/d]	17	0.06	-0.5	1.60	(24.66)	1.61	(24.66)
Beer [g/d]	17	0.06	0.25	35.62	(178.08)	35.62	(178.08)
Butter [g/d]	17	0.06	0	2.89	(10.78)	2.89	(10.78)
Margarine	17	0.06	0.25	11.03	(18.21)	11.03	(18.22)
Other vegetable fat [g/d]	17	0.06	1	2.58	(3.42)	2.58	(3.42)
Other fat [g/d]	17	0.06	-3	0.00	(0.00)	0.00	(0.00)
Fish [g/d]	17	0.06	1	18.36	(19.18)	18.36	(19.18)
Processed meat [g/d]	17	0.06	1	49.44	(46.39)	49.44	(46.41)
Plant oil	17	0.06	1	2.38	(3.37)	2.38	(0.34)
Olive oil	17	0.06	-1	0.30	(1.49)	0.30	(1.49)
Alcohol intake	17	0.06					
0g/day				3.08 %		3.07 %	
>0-6 g/day				39.75 %		39.73 %	
>6-12 g/day				21.16 %		21.15 %	
>12-24 g/day				18.75 %		18.74 %	
>24-60 g/day				14.61 %		14.60 %	
>60 (female)/>60-96 (male) g/d				2.23 %		2.25 %	
>96 g/day				0.43 %		0.46 %	
Highest school level [six-stage]	18	0.07					
current in training				0.06 %		0.06 %	
no certificate				2.59 %		2.59 %	
part skilled worker				0.90 %		0.90 %	
skilled worker				35.13 %		35.12 %	
professional school				24.83 %		24.83 %	
collage of higher education				10.78 %		10.78 %	
university				25.71 %		25.72 %	

[continued on next page]

Parameters included in imputation procedure	Missing values: Number	Missing values: Proportion (%)	Lambda[a]	Median (IQR) or proportion: Before imputation	Median (IQR) or proportion: After imputation
**EPIC-Potsdam (n=27,548)**					
Highest school level [three-stage]	18	0.07			
no vocational training/vocational training				38.67 %	38.66 %
technical college				24.83 %	24.83 %
university				36.49 %	36.51 %
Highest school level [four-stage]	18	0.07			
current in training/no certificate/part skilled worker				3.55 %	3.55 %
skilled worker				35.13 %	35.12 %
professional school				24.83 %	24.83 %
collage of higher education, university				36.49 %	36.50 %
Television watching, h/d, last 12 months	20	0.07	0.25	2.00 (2.00)	2.00 (2.00)
Type 2 diabetes, self-reported at baseline (yes)	23	0.08		4.76 %	4.76 %
Waist circumference [cm]	42	0.15	0.5	86.0 (19.5)	86.0 (19.5)
Waist-Hip-Ratio	50	0.18	1.25	0.84 (0.16)	0.84 (0.16)
Self-reported diabetes duration at baseline [years]	91	0.33		0.00 (0.00)	0.00 (0.00)
Height [cm]	161	0.58	2.25	167.0 (12.4)	167.0 (12.4)
BMI	212	0.77	0.75	25.76 (5.38)	25.78 (5.41)
Weight aged 25 years	1948	7.07	0.25	63.0 (15.0)	63.0 (16.0)
Diastolic blood pressure	1463	5.31	0.75	83.0 (13.5)	82.5 (14.0)
Systolic blood pressure	1470	5.34	-0.75	127.5 (22.0)	127.0 (22.5)
Diabetic father=yes /age of diagnosis	4216	15.30			
no/unknown				89.90 %	89.17 %
yes, at age <30				0.17 %	0.17 %
yes, at age 30-59				2.96 %	2.83 %
yes, at age >=60				6.18 %	6.82 %
yes, age unknown				0.79 %	1.02 %
Diabetic mother=yes /age of diagnosis	4216	15.30			
no/unknown				82.67 %	78.89 %
yes, at age <30				0.12 %	4.41 %
yes, at age 30-59				4.86 %	4.72 %
yes, at age >=60				10.97 %	10.44 %
yes, age unknown				1.38 %	1.54 %

[continued on next page]

Parameters included in imputation procedure	Missing values: Number	Missing values: Proportion (%)	Lambda[a]	Median (IQR) or proportion: Before imputation	Median (IQR) or proportion: After imputation
**EPIC-Potsdam (n=27,548)**					
Diabetic sibling=yes /age of diagnosis	4216	15.30			
no/unknown				94.44 %	92.14 %
yes, at age <30				0.33 %	0.72 %
yes, at age 30-59				3.02 %	4.48 %
yes, at age >=60				1.75 %	1.99 %
yes, age unknown				0.45 %	0.67 %
MI father=yes /age of diagnosis	4216	15.30			
no/unknown				87.59 %	87.15 %
yes, at age <60				4.73 %	5.11 %
yes, at age >=60				7.15 %	7.11 %
yes, age unknown				0.53 %	0.62 %
MI mother=yes /age of diagnosis	4216	15.30			
no/unknown				94.47 %	94.23 %
yes, at age <60				1.10 %	1.26 %
yes, at age >=60				4.16 %	4.22 %
yes, age unknown				0.28 %	0.29 %
MI siblings=yes /age of diagnosis	4216	15.30			
no/unknown				97.06 %	93.99 %
yes, at age <60				1.68 %	2.87 %
yes, at age >=60				1.08 %	1.82 %
yes, age unknown				0.17 %	1.32 %
Stroke father=yes /age of diagnosis	4216	15.30			
no/unknown				91.03 %	88.38 %
yes, at age <60				1.44 %	3.84 %
yes, at age >=60				7.09 %	6.84 %
yes, age unknown				0.44 %	0.94 %

[continued on next page]

Parameters included in imputation procedure	Missing values: Number	Missing values: Proportion (%)	Lambda[a]	Median (IQR) or proportion: Before imputation		Median (IQR) or proportion: After imputation	
**EPIC-Potsdam (n=27,548)**							
Stroke mother=yes /age of diagnosis	4216	15.30					
no/unknown				88.52 %		87.59 %	
yes, at age <60				1.11 %		1.57 %	
yes, at age >=60				9.93 %		10.37 %	
yes, age unknown				0.44 %		0.47 %	
Stroke siblings=yes /age of diagnosis	4216	15.30					
no/unknown				97.81 %		97.42 %	
yes, at age <60				0.94 %		1.19 %	
yes, at age >=60				1.13 %		1.24 %	
yes, age unknown				0.12 %		0.16 %	
Number of brothers and sisters	4701	17.06		1	(1)	1	(2)
Weight aged 40 years	6481	23.53	0	70.0	(19.0)	68.0	(18.0)
Uric acid [mg/dl]	23769	86.28	0.5	4.81	(2.03)	4.65	(1.91)
Calcium [mmol/l]	23769	86.28	3	1.95	(0.14)	1.96	(0.14)
Magnesium [mmol/l]	23769	86.28	1.25	0.81	(0.10)	0.82	(0.09)
Phosphorus [mmol/l]	23769	86.28	1.25	0.88	(0.22)	0.88	(0.22)
Glutamate Pyruvate Transaminase (GPT)[U/L]	23774	86.30	-0.25	22.0	(16.0)	20.0	(13.0)
hsCRP (high-sensitiv CRP) [mg/dl]	23775	86.30	0	0.09	(0.25)	0.07	(0.19)
Triglycerides [mg/dl]	23776	86.31	-0.25	122.08	(105.28)	108.00	(84.10)
Glucose [mg/dl]	23778	86.31	-0.75	104.01	(22.18)	102.56	(18.59)
Total cholesterol [mg/dl]	23780	86.32	0.25	206.93	(54.64)	200.73	(53.37)
HDL cholesterol [mg/dl]	23780	86.32	0	51.64	(19.02)	52.91	(18.09)
Gamma-GT (gamma-glutamyl transferase)[U/L]	23780	86.32	-0.25	21.0	(27.0)	19.0	(21.0)
Adiponectin [ng/ml]	23816	86.45	0	7155.43	(4883.01)	7097.16	(4656.69)
HbA1c (glycated hemoglobin) [%]	23878	86.68	-0.25	5.55	(0.80)	5.46	(0.66)
Insulin [pmol/l]	26945	97.81	-0.25	44.49	(44.58)	39.79	(34.47)

[continued on next page]

Parameters included in imputation procedure	Missing values: Number	Missing values: Proportion (%)	Lambda[a]	Median (IQR) or proportion: Before imputation		Median (IQR) or proportion: After imputation	
**EPIC-Heidelberg (n=25,540)**							
Gender (female)	0	0.00		53.30 %		53.30 %	
Rounded age at baseline	0	0.00		51	(14)	51	(14)
Waist circumference [cm]	0	0.00		88.0	(19.2)	88.0	(19.2)
Body height [cm]	0	0.00		169.0	(12.9)	169.0	(12.9)
Body Mass Index (computed)	0	0.00		25.65	(5.43)	25.65	(5.43)
Wholegrain [50g portion/d]	0	0.00		0.75	(1.44)	0.75	(1.44)
Coffee [150g portion/d]	0	0.00		2.00	(3.29)	2.00	(3.29)
Red meat [150g portion/d]	0	0.00		0.24	(0.26)	0.24	(0.26)
High-energy soft drinks [200ml portion/d]	0	0.00		0.02	(0.17)	0.02	(0.17)
Plant oil [10g portion/d]	0	0.00		0.58	(0.43)	0.58	(0.43)
Other bread [g/d]	0	0.00		76.42	(89.30)	76.42	(89.30)
Fresh fruit [g/d]	0	0.00		91.23	(91.41)	91.23	(91.41)
Canned fruit [g/d]	0	0.00		1.97	(8.88)	1.97	(8.88)
Raw vegetables [g/d]	0	0.00		38.23	(35.45)	38.23	(35.45)
Cooked vegetables [g/d]	0	0.00		25.23	(21.52)	25.23	(21.52)
Legumes [g/d]	0	0.00		12.65	(17.95)	12.65	(17.95)
Fried potatoes [g/d]	0	0.00		17.42	(22.10)	17.42	(22.10)
Nuts [g/d]	0	0.00		0.82	(3.70)	0.82	(3.70)
Low-fat dairy products [g/d]	0	0.00		6.16	(55.90)	6.16	(55.90)
High-fat dairy products [g/d]	0	0.00		53.76	(139.25)	53.76	(139.25)
Low-fat cheese [g/d]	0	0.00		0	(2.02)	0	(2.02)
High-fat cheese [g/d]	0	0.00		16.57	(27.85)	16.57	(27.85)
Fruit juice [g/d]	0	0.00		88.37	(184.10)	88.37	(184.10)
Low-energy soft drinks [g/d]	0	0.00		0	(0)	0	(0)
High-energy soft drinks [g/d]	0	0.00		3.70	(33.72)	3.70	(33.72)
Beer [g/d]	0	0.00		41.10	(173.97)	41.10	(173.97)
Butter [g/d]	0	0.00		5.47	(11.21)	5.47	(11.21)
Margarine	0	0.00		0.36	(5.00)	0.36	(5.00)
Other vegetable fat [g/d]	0	0.00		4.50	(4.05)	4.50	(4.05)
Other fat [g/d]	0	0.00		0	(0)	0	(0)
Fish [g/d]	0	0.00		15.89	(21.53)	15.89	(21.53)
Processed meat [g/d]	0	0.00		36.79	(40.47)	36.79	(40.47)
MI case after 10 years (yes)	0	0.00		1.52 %		1.52 %	

[continued on next page]

Parameters included in imputation procedure	Missing values: Number	Missing values: Proportion (%)	Lambda[a]	Median (IQR) or proportion: Before imputation		Median (IQR) or proportion: After imputation	
**EPIC-Heidelberg (n=25,540)**							
Stroke case after 10 years (yes)	0	0.00		1.46 %		1.46 %	
Diabetes case after 10 years (yes)	216	0.85		8.22 %			
Nelson-Aalen estimate for MI follow-up	0	0.00		0.0172928	(0)	0.0172928	(0)
Nelson-Aalen estimate for stroke follow-up	0	0.00		0.0167023	(0)	0.0167023	(0)
Nelson-Aalen estimate for T2D follow-up	0	0.00		0.1004579	(0)	0.1004579	(0)
Member of subcohort (yes)	0	0.00		10.72 %		10.72 %	
Type 2 diabetes, self-reported at baseline (yes)	5	0.02		3.44 %		3.46 %	
Hypertension, self-reported at baseline (yes	30	0.12		28.74 %		28.74 %	
Smoking Status	1038	4.06					
Never				41.36 %		41.35 %	
Former [<20 units/d]				19.34 %		19.45 %	
Former [≥20 units/d]				15.08 %		15.10 %	
Current [<20 units/d]				14.10 %		14.09 %	
Current [≥20 units/d]				10.13 %		10.00 %	
Physical activity [h/week]	1384	5.42	0	2.25	(3.00)	2.25	(3.00)
Parents with CVD	7258	28.42					
None				52.48 %		51.36 %	
One				39.66 %		40.75 %	
Two				7.87 %		7.89 %	
Parents with type 2 diabetes	7678	30.06					
None				75.50 %		71.94 %	
One				22.19 %		25.32 %	
Both				2.32 %		2.74 %	
MI in first degree relatives (yes)	10089	39.50		37.93 %		37.81 %	
At least one sibling with CVD (yes)	10275	40.23		12.02		13.12 %	
At least one sibling with diabetes (yes)	10320	40.41		9.97 %		12.39 %	
Systolic blood pressure [mmHg]	15249	59.71	-0.25	126.5	(22.0)	127.0	(22.5)
Diastolic blood pressure [mmHg]	15249	59.71	0.25	82.0	(14.5)	82.5	(14.5)
HbA1c [%]	22388	87.66	-1	5.35	(0.46)	5.35	(0.46)
HDL cholesterol [mg/dl]	22533	88.23	-0.25	54.14	(23.20)	54.14	(19.34)
Total cholesterol [mg/dl]	22877	89.57	0.5	228.15	(54.14)	228.15	(54.14)
Triglycerides [mg/dl]	22882	89.59	-0.25	141.71	(115.14)	141.71	(106.28)

[a] Lambda used for BoxCox-Transformation before imputation

Supplementary Table 2 Sensitivity, specificity, positive predictive value (PPV), and negative predictive value (NPV) of the derived non-clinical and clinical score in EPIC-Potsdam and EPIC-Heidelberg.

	EPIC-Potsdam		EPIC-Heidelberg	
	Non-clinical score	Clinical score	Non-clinical score	Clinical score
**Cut-off: 5%**				
Sensitivity [%]	49.1	48.1	48.8	53.3
Specificity [%]	87.0	87.7	83.4	81.9
PPV [%]	8.0	8.2	8.2	8.1
NPV [%]	98.7	98.7	98.2	98.3
**Cut-off: 7.5%**				
Sensitivity [%]	28.8	29.1	31.1	35.8
Specificity [%]	93.8	94.0	91.7	90.2
PPV [%]	9.5	10.1	10.1	10.0
NPV [%]	98.3	98.3	97.8	97.9
**Cut-off: 10%**				
Sensitivity [%]	17.0	17.7	18.8	24.3
Specificity [%]	96.6	96.7	95.5	94.3
PPV [%]	10.1	11.1	11.2	11.6
NPV [%]	98.1	98.1	97.5	97.6

Supplementary Table 3 Risk association of the predictors included in the developed scores with cardiovascular events in other meta-analyses or large-scale studies. Effect estimates are shown with according 95% confidence intervals (CI) if not indicated otherwise

Predictor	Study	Design	Population	Exposure	Outcome	Estimate, 95% CI (if not indicated otherwise)
Gender	George et al 2015 [7]	Cohort (linked electronic health records)	n= 1,937,360	male	Ischemic stroke<br>MI<br>Intracerebral Haemorrhage	HR 1.62, 1.49-1.78<br>HR 3.72, 3.53-3.91<br>HR 1.39, 1.23-1.58
Waist circumference	Wormser et al 2011 [8]	individual records from 58 cohorts	n= 221,934	Increase per standard deviation (12.26 cm)	CVD<br>CHD<br>Ischemic stroke	HR 1.10, 1.05-1.14<br>HR 1.12, 1.06-1.19<br>HR 1.11, 1.05-1.17
Smoking	Mons et al 2015 [9]	Meta-analysis	25 cohorts	Former smokers<br><br>Current smokers<br><br>Heavy current (≥20 units daily)	Coronary events<br>Stroke<br>Coronary events<br>Stroke<br>Coronary events<br>Stroke	HR 1.18, 1.06-1.32<br>HR 1.17, 1.07-1.26<br>HR 1.98, 1.75-2.25<br>HR 1.58, 1.40-1.78<br>HR 2.43, 2.01-2.93<br>HR 1.91, 1.66-2.21
Hypertension	Yusuf et al 2004 [10]	Case-control study	15,152 cases<br>14,820 controls	history of hypertension	MI	OR 1.91, 99%CI 1.74-2.10
Diabetes	Sarwar et al 2010 [11]	Meta-analysis	n= 698,782	prevalent T2D	Overall CHD<br>Fatal CHD<br>Non-fatal CHD<br>Ischemic stroke<br>Haemorrhagic	HR 2.00, 1.83-2.19<br>HR 2.31, 2.05-2.60<br>HR 1.82, 1.64-2.03<br>HR 2.27, 1.95-2.65<br>HR 1.56, 1.19-2.05
Family history	Sesso et al 2001 [12]	Prospective cohort (PHS, WHS)	22,071 men<br>39,876 women	Paternal MI<br>Maternal MI<br>Both	CVD	RR men 1.71; women 1.46<br>RR men 1.46; women 1.15<br>RR men 1.85; women 2.05
	Bachmann et al 2012 [13]	Prospective cohort	49,255 men	family history of angina, MI, angioplasty, or coronary artery bypass surgery in sibling, aunt or uncle, parent, or grandparent	CVD mortality	HR 1.51, 0.96–2.39 premature<br>HR 1.40, 1.06–1.86 (>50 years)
	Kasiman et al 2014 [14]	Population-based cohort	n= 62,766<br>n= 31,659<br>unexposed<br>(n= 143,728; 265,974)	sibling history of MI<br>sibling history of ischemic stroke	Stroke<br>MI	RR 1.41, 1.32-1.50<br>RR 1.44, 1.34-1.55

[continued on next page]

Predictor	Study	Design	Population	Exposure	Outcome	Estimate, 95% CI (if not indicated otherwise)
Whole grain	Aune et al 2016 [15]	meta-analysis	CVD n= 704,317<br>CHD n= 316,491<br>Stroke n= 245,012	Per 90g/day increment in consumption	CVD<br>CHD<br>Stroke	HR 0.78, 0.73-0.85<br>HR 0.81, 0.75-0.87<br>HR 0.88, 0.75-1.03
	Bechthold et al 2017 [16]	meta-analysis		per intake of 30g/day	CHD<br>Stroke	RR 0.95, 0.92-0.98<br>RR 0.99, 0.95-1.03
Red meat	Bechthold et al 2017 [16]	meta-analysis		Per intake of 100g/d	CHD<br>Stroke	RR 1.15, 1.08- 1.23<br>RR 1.12, 1.06- 1.17
Coffee	Poole et al 2017 [17]	umbrella-review of multiple meta-analyses		3 cups/day compared with non-drinkers	CVD mortality<br>CVD morbidity	RR 0.81, 0.72-0.90<br>RR 0.85, 0.80-0.90
High-energy soft drinks	Bechthold et al 2017 [16]	meta-analysis		Per additional 250ml/day	CHD<br>Stroke	RR 1.17, 1.11-1.23<br>RR 1.07, 1.02-1.12
Plant oil	Abdelhamid et al 2020 [18]	meta-analysis of intervention studies	n= 140,482<br>n= 134,116<br>n= 138,888	dietary omega-3 FA (low vs high)	CVD<br>CHD<br>Stroke	RR 0.96, 0.92-1.01<br>RR 0.91, 0.85-0.97<br>RR 1.02, 0.94-1.12
	Hooper et al 2018 [19]	meta-analysis of intervention studies	n= 4,962<br>n= 3,997<br>n= 3,730	dietary omega-6 FA (low vs high)	CVD<br>CHD<br>Stroke	RR 0.97, 0.81-1.15<br>RR 0.88, 0.66-1.17<br>RR 1.36, 0.45-4.11
	Martinez-Gonzalez et al 2014 [20]	meta-analysis	n= 101,460 CHD<br>n= 38,673 stroke	per 25g increase in daily olive oil consumption	CHD<br>CHD + stroke	RR 0.76, 0.67-0.86<br>RR 0.82, 0.70-0.96
Systolic BP	Singh et al 2013 [21]	meta-analysis	123 cohorts	per 10 mmHg	Ischemic heart disease<br>Ischemic stroke	age group 35-44: RR 1.68, 1.29-2.20<br>age group 65-74: RR 1.33, 1.29-1.38<br>age group 35-44: RR 2.05, 1.89-2.22<br>age group 65-74: RR 1.44, 1.39-1.50

[continued on next page]

Predictor	Study	Design	Population	Exposure	Outcome	Estimate, 95% CI (if not indicated otherwise)
Diastolic BP	Lewington et al 2002 [22]	meta-analysis	61 cohorts	per 10 mmHg	Mortality of ischemic Heart disease<br>Mortality of stroke	age group 50-59: HR 0.52, 0.50-0.55<br>age group 50-59: HR 0.34, 0.32-0.37
Cholesterol	Singh et al 2013 [21]	meta-analysis	123 cohorts	per 1 mmol/L	Ischemic heart disease<br>Ischemic stroke	age group 35-44: RR 2.22, 1.46-3.33<br>age group 65-74: RR 1.27, 1.23-1.32<br>age group 35-44: RR 1.71, 1.46-1.99<br>age group 65-74: RR 1.08, 1.03-1.13
HDL cholesterol	Emerging Risk Factors et al. 2009 [23]	meta-analysis	n= 302,430	Per 15mg/dl	CHD<br>Ischemic stroke<br>Haemorrhagic stroke	HR 0.71, 0.68-0.75<br>HR 0.93, 0.84-1.02<br>HR 1.09, 0.92-1.29

HR, hazard ratio. CI, confidence interval. RR, relative risk. OR, odds ratio. CVD, cardiovascular disease. CHD, coronary heart disease. MI, myocardial infarction. HDL, high density lipoprotein.

Supplementary Table 4 Selected food groups derived from the Food Frequency Questionnaire (FFQ) in EPIC-Potsdam and EPIC-Heidelberg and the according summarised food items.

Food group	Summarised items
Whole grain products	Whole grain bread, dark and whole grain rolls;
	Grain flakes, grains, muesli
Nuts	Nuts
Coffee	Coffee with caffeine (black, with milk, with condensed milk, with sweeteners)
High-energy soft drinks	Cola, lemonade (normal), alcohol free beer, malt beer
Plant oil	Olive oil (with meat/fish, vegetables, as salad dressing), plant oil (excluding coconut fat) for cooking (with meat/fish, vegetables), sunflower and seed oil, other oil (with meat/fish, vegetables), sunflower and safflower oil, other oil (as salad dressing)
Fish	Canned fish, smoked fish, fish (filet, fish fingers)
Red meat	Pork schnitzel, pork cutlet, steak, filet, roast pork, pork goulash, diced pork, Kassler, spare rib, boiled pork meat, knuckle of pork, pork belly, ground beef, meat loaf, minced meat sauce, hash, liver, calf and lamb meat, rabbit, steak, filet and loin from beef, roast beef, boiled beef, beef roulade, beef goulash, diced beef
Vegetables	Raw vegetables: cucumber, radish, red radish, cabbage salad, carrots, sprouts, bell peppers, capsicum, tomato, raw onions (with salad), lettuce, endive, lamb's lettuce, Chinese cabbage, mixed salad
	Cooked vegetables: hot tomato chutney, tomato sauce, cooked bell peppers, zucchini, eggplant, spinach, leek, carrots, comfrey, celery, asparagus, peas and carrots mixed vegetables, sauerkraut
Fruits	Apple, pear, peach, nectarine, cherries, plums, mirabelles, grapes, strawberries, currant, raspberries, blackberries, banana, kiwi, mango, fresh pineapple, oranges, grapefruit, mandarin

Supplementary Table 5 Number of participants of the overall EPIC-Potsdam and EPIC-Heidelberg cohorts, the randomly drawn subcohort, cardiovascular disease (CVD), type 2 diabetes (T2D), and transient ischemic attack (TIA) cases respectively up to the time point of sampling and the according overlap with the subcohort.

	EPIC-Potsdam	EPIC-Heidelberg
Overall cohort n	27,548	25,540
Subcohort n	2,500	2,739
T2D cases n	820	---
(n internal cases among these)	(74)	
CVD cases n	508	917
(n internal cases among these)	(62)	(172)
TIA cases n	239	---
(n internal cases among these)	(21)	

Supplementary Table 6 Parameters and according equations used for calculation of absolute risks with external scores for comparison.

Parameter	Recalibrated pooled cohort equation [a] (De las Heras Gala, 2016, Suppl)		Framingham Risk Score including blood lipids [a] (D'Agostino, 2008)		Framingham Risk Score including BMI [a] (D'Agostino, 2008)		SCORE for low risk countries[b] (Conroy, 2003)	
**β coefficients**	Women	Men	Women	Men	Women	Men	CHD	Non-CHD
Log(age)	-29.799	12.344	2.32888	3.06117	2.72107	3.11296		
Log(age)^2	+4.884	---	---	---	---	---		
Log (cholesterol)	13.540	+11.853	+1.20904	+1.12370	---	---		
Log(age)*log(cholesterol)	-3.114	-2.664	---	---	---	---		
Log(HDL cholesterol)	-13.578	-7.990	-0.70833	-0.93263	---	---		
Log(age)*log(HDL cholesterol)	+3.149	+1.769	---	---	---	---		
Log(treated systolic blood pressure)	+2.019	+1.797	+2.82263	+1.99881	+2.88267	1.92672		
Log(untreated systolic blood pressure)	+1.957	+1.764	+2.76157	+1.93303	+2.81291	1.85508		
Current smoking (yes)	+7.574	+7.837	+0.52873	+0.65451	+0.61868	0.70953	0.71	0.63
Log(age)*current smoking	-1.665	-1.795	--	---	---	---		
Prevalent diabetes (yes)	+0.661	+0.658	+0.69154	+0.57367	+0.77763	0.53160		
Log(BMI)	---	---	---	---	+0.51125	0.79277		
Cholesterol (mmol/l)							0.24	0.02
Systolic blood pressure (mmHg)							0.018	0.022
**Cox parameters**								
Mean points $\sum_{i=1}^{p} \beta_i \overline{X_i}$	-29.18	61.18	26.1931	23.9802	26.0145	23.9388		
Baseline risk S_0	0.9665	0.9144	0.95012	0.88936	0.94833	0.88431		
Correction factor	-0.434997	-0.434997	---	---	---	---		
**Weibull parameters**								
**α coefficients (scale)**								
Low risk men							-22.1	-26.7
Low risk women							-29.8	-31.0
**p coefficients (shape)**								
Low risk men							4.71	5.64
Low risk women							6.36	6.62

CHD, coronary heart disease. SCORE, Systematic COronary Risk Evaluation.

We used the provided risk charts to calculate score points for discrimination assessment of the chronic metabolic disease risk score (Alssema, 2012), PROCAM for myocardial infarction (Assmann, 2002), and PROCAM for stroke (Assmann, 2007). We categorized parameters according to the chart and summarized the assigned points.

[a] Based on Cox proportional hazards regression models. Absolut risks were calculated according to the published equation:

$$\hat{p} = 1 - S_0(t)^{\exp(\sum_{i=1}^{p} \beta_i X_i - \sum_{i=1}^{p} \beta_i \overline{X_i})}$$

[b] Based on a Weibull model with age as underlying timescale. Absolut risks were calculated according to the published equations:

$$S_0(age) = exp\{-(\exp(\alpha))(\text{age} - 20)^p\}$$

$$S_0(age + 10) = exp\{-(\exp(\alpha))(\text{age} - 10)^p\}$$

$$w = \beta_{chol}(cholesterol - 6) + \beta_{SBP}(SBP - 120) + \beta_{smoker}(current)$$

$$S(age) = \{S_0(age)\}^{exp(w)}$$

$$S(age + 10) = \{S_0(age + 10)\}^{exp(w)}$$

$$S_{10}(age) = S(age + 10)/S(age)$$

$$Risk_{10} = 1 - S_{10}(age)$$

$$CVDRisk_{10}(age) = [CHDRisk(age)] + [Non - CHDRisk(age)]$$

References

1. Kroke A, Bergmann MM, Lotze G, Jeckel A, Klipstein-Grobusch K, Boeing H. Measures of quality control in the German component of the EPIC study. European Prospective Investigation into Cancer and Nutrition. Ann Nutr Metab. 1999;43(4):216-24. doi:10.1159/000012788.
2. White IR, Royston P, Wood AM. Multiple imputation using chained equations: Issues and guidance for practice. Statistics in medicine. 2011;30(4):377-99. doi:10.1002/sim.4067.
3. Osborne J. Improving your data transformations: Applying the Box-Cox transformation

Practical Assessment, Research & Evaluation. 2010;15(12):1-9.
4. Schenker N, Taylor JMG. Partially parametric techniques for multiple imputation. Computational Statistics & Data Analysis. 1996;22(4):425-46.
5. Rubin DB. Multiple Imputation for Nonresponse in Surveys. Wiley; 2004.
6. Marshall A, Altman DG, Holder RL, Royston P. Combining estimates of interest in prognostic modelling studies after multiple imputation: current practice and guidelines. BMC medical research methodology. 2009;9:57. doi:10.1186/1471-2288-9-57.
7. George J, Rapsomaniki E, Pujades-Rodriguez M, Shah AD, Denaxas S, Herrett E et al. How Does Cardiovascular Disease First Present in Women and Men? Incidence of 12 Cardiovascular Diseases in a Contemporary Cohort of 1,937,360 People. Circulation. 2015;132(14):1320-8. doi:10.1161/circulationaha.114.013797.
8. Wormser D, Kaptoge S, Di Angelantonio E, Wood AM, Pennells L, Thompson A et al. Separate and combined associations of body-mass index and abdominal adiposity with cardiovascular disease: collaborative analysis of 58 prospective studies. Lancet. 2011;377(9771):1085-95. doi:10.1016/s0140-6736(11)60105-0.
9. Mons U, Müezzinler A, Gellert C, Schöttker B, Abnet CC, Bobak M et al. Impact of smoking and smoking cessation on cardiovascular events and mortality among older adults: meta-analysis of individual participant data from prospective cohort studies of the CHANCES consortium. BMJ : British Medical Journal. 2015;350:h1551. doi:10.1136/bmj.h1551.
10. Yusuf S, Hawken S, Ounpuu S, Dans T, Avezum A, Lanas F et al. Effect of potentially modifiable risk factors associated with myocardial infarction in 52 countries (the INTERHEART study): case-control study. Lancet. 2004;364(9438):937-52. doi:10.1016/s0140-6736(04)17018-9.
11. Sarwar N, Gao P, Seshasai SR, Gobin R, Kaptoge S, Di Angelantonio E et al. Diabetes mellitus, fasting blood glucose concentration, and risk of vascular disease: a collaborative meta-analysis of 102 prospective studies. Lancet. 2010;375(9733):2215-22. doi:10.1016/s0140-6736(10)60484-9.
12. Sesso HD, Lee IM, Gaziano JM, Rexrode KM, Glynn RJ, Buring JE. Maternal and paternal history of myocardial infarction and risk of cardiovascular disease in men and women. Circulation. 2001;104(4):393-8. doi:10.1161/hc2901.093115.
13. Bachmann JM, Willis BL, Ayers CR, Khera A, Berry JD. Association between family history and coronary heart disease death across long-term follow-up in men: the Cooper Center Longitudinal Study. Circulation. 2012;125(25):3092-8. doi:10.1161/circulationaha.111.065490.
14. Kasiman K, Lundholm C, Sandin S, Malki N, Sparen P, Ingelsson E. Common Familial Effects on Ischemic Stroke and Myocardial Infarction: A Prospective Population-Based Cohort Study. Frontiers in cardiovascular medicine. 2014;1:3. doi:10.3389/fcvm.2014.00003.
15. Aune D, Keum N, Giovannucci E, Fadnes LT, Boffetta P, Greenwood DC et al. Whole grain consumption and risk of cardiovascular disease, cancer, and all cause and cause specific mortality: systematic review and dose-response meta-analysis of prospective studies. Bmj. 2016;353:i2716. doi:10.1136/bmj.i2716.
16. Bechthold A, Boeing H, Schwedhelm C, Hoffmann G, Knuppel S, Iqbal K et al. Food groups and risk of coronary heart disease, stroke and heart failure: A systematic review and dose-response meta-analysis of prospective studies. Critical reviews in food science and nutrition. 2017:1-20. doi:10.1080/10408398.2017.1392288.
17. Poole R, Kennedy OJ, Roderick P, Fallowfield JA, Hayes PC, Parkes J. Coffee consumption and health: umbrella review of meta-analyses of multiple health outcomes. Bmj. 2017;359:j5024. doi:10.1136/bmj.j5024.
18. Abdelhamid AS, Brown TJ, Brainard JS, Biswas P, Thorpe GC, Moore HJ et al. Omega-3 fatty acids for the primary and secondary prevention of cardiovascular disease. Cochrane Database Syst Rev. 2020;3:Cd003177. doi:10.1002/14651858.CD003177.pub5.
19. Hooper L, Al-Khudairy L, Abdelhamid AS, Rees K, Brainard JS, Brown TJ et al. Omega-6 fats for the primary and secondary prevention of cardiovascular disease. Cochrane Database Syst Rev. 2018;11:Cd011094. doi:10.1002/14651858.CD011094.pub4.
20. Martinez-Gonzalez MA, Dominguez LJ, Delgado-Rodriguez M. Olive oil consumption and risk of CHD and/or stroke: a meta-analysis of case-control, cohort and intervention studies. The British journal of nutrition. 2014;112(2):248-59. doi:10.1017/s0007114514000713.
21. Singh GM, Danaei G, Farzadfar F, Stevens GA, Woodward M, Wormser D et al. The age-specific quantitative effects of metabolic risk factors on cardiovascular diseases and diabetes: a pooled analysis. PLoS One. 2013;8(7):e65174. doi:10.1371/journal.pone.0065174.

22. Lewington S, Clarke R, Qizilbash N, Peto R, Collins R. Age-specific relevance of usual blood pressure to vascular mortality: a meta-analysis of individual data for one million adults in 61 prospective studies. Lancet. 2002;360(9349):1903-13. doi:10.1016/s0140-6736(02)11911-8.
23. Emerging Risk Factors C, Di Angelantonio E, Sarwar N, Perry P, Kaptoge S, Ray KK et al. Major lipids, apolipoproteins, and risk of vascular disease. JAMA. 2009;302(18):1993-2000. doi:10.1001/jama.2009.1619.
